# The Critical Role of Notch Ligand Delta-like 1 in the Pathogenesis of Influenza A Virus (H1N1) Infection

**DOI:** 10.1371/journal.ppat.1002341

**Published:** 2011-11-03

**Authors:** Toshihiro Ito, Ronald M. Allen, William F. Carson, Matthew Schaller, Karen A. Cavassani, Cory M. Hogaboam, Nicholas W. Lukacs, Akihiro Matsukawa, Steven L. Kunkel

**Affiliations:** 1 Department of Pathology, University of Michigan Medical School, Ann Arbor, Michigan, United States of America; 2 Department of Pathology and Experimental Medicine, Graduate School of Medicine, Dentistry and Pharmaceutical Sciences, Okayama University, Okayama, Japan; University of North Carolina at Chapel Hill, United States of America

## Abstract

Influenza A viral infections have been identified as the etiologic agents for historic pandemics, and contribute to the annual mortality associated with acute viral pneumonia. While both innate and acquired immunity are important in combating influenza virus infection, the mechanism connecting these arms of the immune system remains unknown. Recent data have indicated that the Notch system is an important bridge between antigen-presenting cells (APCs) and T cell communication circuits and plays a central role in driving the immune system to overcome disease. In the present study, we examine the role of Notch signaling during influenza H1N1 virus infection, focusing on APCs. We demonstrate here that macrophages, but not dendritic cells (DCs), increased Notch ligand Delta-like 1 (Dll1) expression following influenza virus challenge. Dll1 expression on macrophages was dependent on retinoic acid-inducible gene-I (RIG-I) induced type-I IFN pathway, and not on the TLR3-TRIF pathway. We also found that IFNα-Receptor knockout mice failed to induce Dll1 expression on lung macrophages and had enhanced mortality during influenza virus infection. Our results further showed that specific neutralization of Dll1 during influenza virus challenge induced higher mortality, impaired viral clearance, and decreased levels of IFN-γ. In addition, we blocked Notch signaling by using γ-secretase inhibitor (GSI), a Notch signaling inhibitor. Intranasal administration of GSI during influenza infection also led to higher mortality, and higher virus load with excessive inflammation and an impaired production of IFN-γ in lungs. Moreover, Dll1 expression on macrophages specifically regulates IFN-γ levels from CD4^+^and CD8^+^T cells, which are important for anti-viral immunity. Together, the results of this study show that Dll1 positively influences the development of anti-viral immunity, and may provide mechanistic approaches for modifying and controlling the immune response against influenza H1N1 virus infection.

## Author Summary

Influenza viruses cause annual epidemics and occasional pandemics that have claimed the lives of millions. Both innate and acquired immunity are essential for protection against influenza virus, and Notch and Notch ligands provide a key bridge between innate and acquired immunity. However, the role of Notch system during influenza virus infection is unknown. Here, we show that Notch ligand Delta-like 1 (Dll1) expression was up-regulated in influenza virus H1N1 challenged macrophages, and was dependent on both retinoic-acid–inducible protein I (RIG-I) and IFNα receptor (IFNαR)-mediated pathways. IFNαR-deficient mice challenged with influenza virus in vivo also display a profoundly impaired Dll1 expression with increased mortality and abrogated IFN-γ production. Treatment of WT mice during influenza infection, with either neutralizing antibodies specific for Dll1 or a γ-secretase inhibitor (GSI), which blocks Notch signaling, resulted in increased mortality, impaired viral clearance, and lower IFN-γ production. In addition, Dll1 specifically regulated IFN-γ production from both CD4^+^and CD8^+^T cells in vitro. Together, these results suggest that Notch signaling through macrophage-dependent Dll1 is critical in providing an anti-viral response during influenza infection by linking innate and acquired immunity.

## Introduction

Influenza virus type A causes acute respiratory infections that are highly contagious and cause significant morbidity and mortality in humans and animals [1], [2]. In 2009, the influenza pandemic caused by the current H1N1 virus affected all the continents of the world [3]. In the United States alone, the 2009 H1N1 influenza virus affected 57 million Americans, with more than 11,000 deaths (CDC report; http://www.cdc.gov/h1n1flu/estimates_2009_h1n1.htm). Although vaccines and other antiviral approaches to control influenza recently have been developed, the disease is by no means under control since these treatments are not available worldwide and their efficacy is less than optimal [1], [4]. Thus, a better understanding of the molecular mechanisms of pathogenesis and of the host immune response to influenza virus infection is required for the prevention and treatment of influenza.

A viral infection is initially sensed by the host innate system, triggering a rapid antiviral response that involves the release of proinflammatory cytokines, and eventually leads to the activation of the adaptive immune response [5]. The first line of defense is initiated when cellular pathogen recognition receptors (PRRs) recognize pathogen-associated molecular patterns (PAMPs) including influenza virus [6], [7]. In many PAMPs, RNA virus is recognized not only by PRR Toll-like receptor 3 (TLR3) but also by RIG-I and melanoma-differentiation-associated gene 5 (MDA5) [8]. During the life cycle of influenza virus, these proteins in turn activate the TBK1 and IKKi kinases, which phosphorylate interferon-regulatory factor-3 (IRF-3) and IRF-7, transcription factors essential for the expression of type-I IFNs [9].

The type-I IFN (IFN-α/β) cytokines are vital to the innate immune response and control the expression of>100 gene products, several of which directly reduce viral replication and spreading by conferring the so-called “antiviral state” [10]. IFN-αβ activates these downstream processes by initially engaging the IFN-α receptor (IFNαR) and activating the JAK-STAT pathway [11]. This pathway induces a number of early-response, IFN-stimulated genes (ISGs) including type II IFN (IFN-γ) [12]. Furthermore, IFN-αβ also activates NFκB, which amplifies the IFN response via a positive-feedback loop. This feedback is important for the recruitment of specialized immune cells to the site of injury or viral infection [6]. IFN-αβ is initially produced by leukocytes and fibroblasts, leading to the recruitment of T and NK cells, that produce IFN-γ. IFN-γ induces and activates numerous key antiviral factors, most notably PKR (RNA-activated protein kinase), a serine/threonine kinase induced by both IFN type I and type II stimulation [12], [13]. Thus, IFN-αβ and IFN-γ affect the activities of macrophages, T cells, dendritic cells (DCs), and NK cells by enhancing antigen presentation, cell trafficking, and cell differentiation profiles, which ultimately enhances antiviral effector functions [13].

In the last decade, it has been demonstrated that Notch signaling pathways contribute to both the hematopoietic and immune systems including a role in the development of embryonic hematopoietic stem cells and a role in multiple lineage decisions of developing lymphoid and myeloid cells [14]. There are 5 mammalian ligands (Delta-like [Dll]1, Dll3, Dll4, Jagged-1, and Jagged-2), each of which can activate any of the 4 Notch receptors (Notch1, -2, -3, -4) [15]. Notch signaling during lymphoid development has been extensively studied, and its essential role in specifying cell fate at many stages during T-cell development is well characterized [14]. Moreover, recent data have indicated that the Notch signaling pathway is an important modulator of Tcell-mediated immune responses [15]. For example, Notch signaling is associated with the differentiation of naive CD8^+^Tcells to cytotoxic T lymphocytes (CTLs), and cytotoxic CD8^+^T are recruited to kill virus-infected cells by the production of IFN-γ [14]. Another function that was assigned to Notch is the regulation of T helper (Th) cell differentiation. The importance of Notch activation has been supported using GSI, which is a pharmacologic inhibitor of Notch signaling pathways, to block the induction of Th1-type responses [14]. Upon recognition of pathogens and presentation of antigen via MHC class II proteins by antigen-presenting cells (APCs) such as macrophages and DCs, CD4^+^Th cells become activated, drive adaptive immunity and induce specific responses to invading microbes [14]. For instance, Th1 cell induction by forced Dll expression on the surface of APCs was shown to induce Th1 cell differentiation, and Dll ligands were thought to inhibit Th2 cell differentiation by interfering with IL-4 receptor signaling [14]. On the other hand, expression of Jagged ligands, but not Dll, on the surface of APCs was shown to induce Th2 cell differentiation [14]. Further, we have demonstrated that Dll4 induction on DCs can specifically promote the generation of Th17 cells [16].

In the present study, we examine the role of Notch signaling during influenza H1N1 virus infection, focusing on APCs because of their central role in driving the immune system to overcome disease. We demonstrate that macrophages, but not DCs, increased Notch ligand Dll1 expression following influenza virus stimulation. Dll1 expression on bone marrow-derived macrophages (BMDMs) was dependent on RIG-I induced type-I IFN pathway, and not on the TLR3-TRIF pathway. We also found that IFNαR^−/−^ mice failed to induce Dll1 expression on lung macrophages and had enhanced mortality during influenza virus infection. Our results further showed that specific neutralization of Dll1 during treatment with a Notch signaling inhibitor during influenza virus challenge induced higher mortality, impaired viral clearance, and decreased levels of IFN-γ. Together, the results of this study show that Dll1 positively influences the development of anti-viral immunity, and may provide mechanistic approaches for modifying and controlling the immune response against influenza H1N1 virus infection.

## Results

### Macrophages, but not DCs, exhibited increased expression level of Dll1

**Figure 1:**
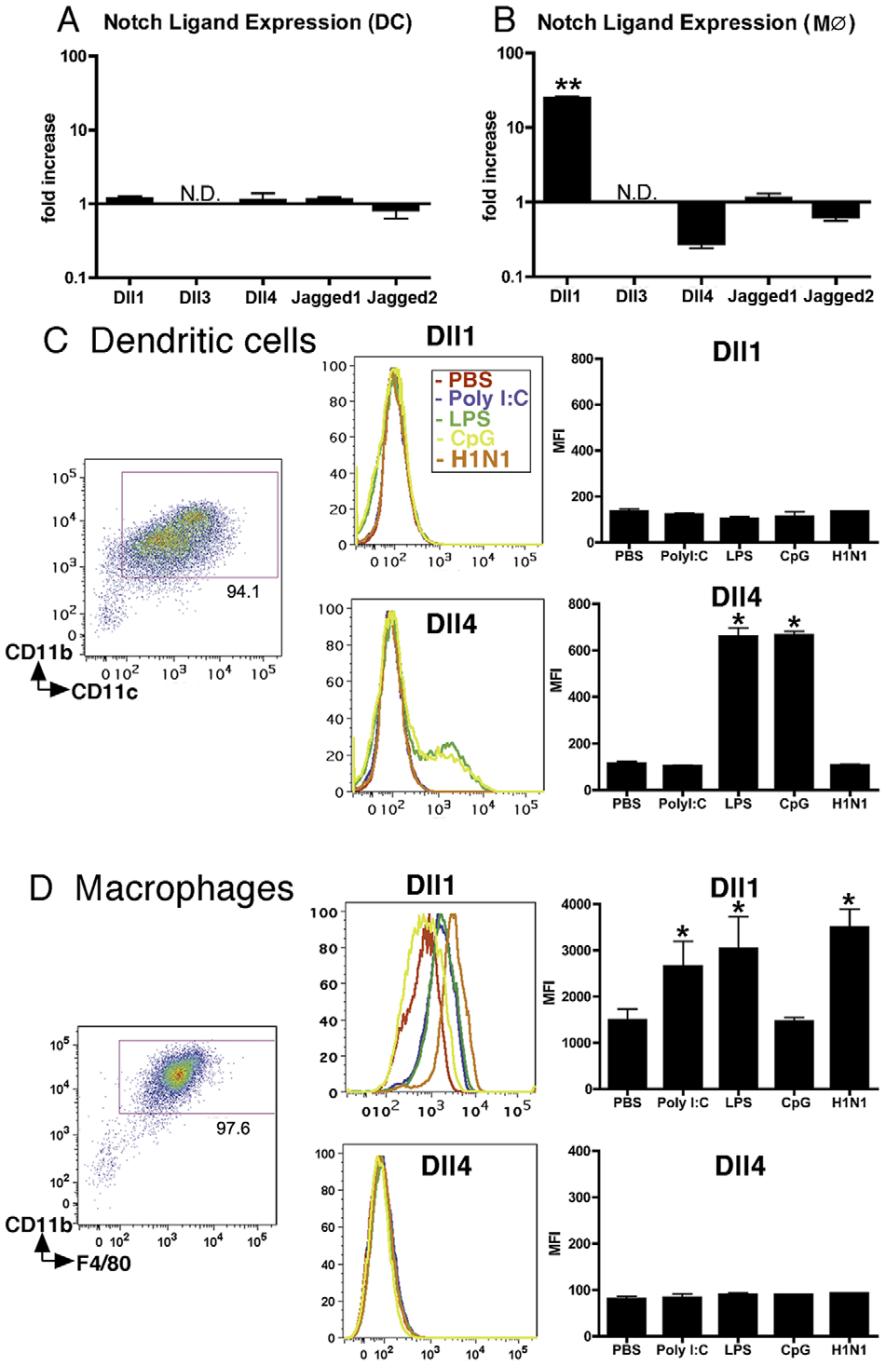
BMDMs, but not BMDCs, exhibit increased expression of Dll1. BM-derived DCs (BMDCs) (**A**) and BMDMs (M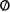) (**B**) were stimulated with H1N1 (MOI = 10) for 6 hours, then quantitative real-time PCR was performed and the expression levels of Notch ligands were evaluated. BMDC (CD11b^+^CD11c^+^) (**C**) and BMDM (CD11b^+^F4/80^+^) (**D**) were stimulated with PolyI:C (10 µg/ml), LPS (1 μg/ml), CpG (1 µM), or H1N1 (MOI = 10) for 24 hours, then flow cytometry was performed as indicated in MATERIALS and METHODS. *P<0.05, **P<0.01 compared with PBS-treated cells. Data shown indicate mean±SEM and are from a representative experiment of 3 independent experiments. Each time point represents at least 4 mice per group. N.D. = No Detection, MFI = Mean Fluorescence Intensity.

Since we previously demonstrated that Dll4 was upregulated on BM-derived DCs (BMDCs) following exposure to certain bacterial antigens including CpG (TLR9 ligand) and BCG [16], we first assessed the gene expression profile of Notch ligands on APCs following influenza virus stimulation. During H1N1 stimulation no Notch ligands were induced on BMDCs (**Fig. 1A**), while Dll1 mRNA levels were increased in BMDMs (**Fig. 1B**). Dll3 expression was below detection levels of our assay. In agreement with the data from BMDMs, H1N1 induced the expression of Dll1 on RAW264.7 cells, a mouse leukemic monocyte macrophage cell line (**Fig. S1**). We next examined protein levels of Notch ligands following treatment with various TLR ligands. No TLR ligands induced expression of Dll1 on BMDCs (CD11b^+^CD11c^+^) (**Fig. 1C**). Though H1N1 failed to induce Dll4 on BMDCs, Dll4 expression was induced on BMDCs following LPS (TLR4 ligand) and CpG (TLR9 ligand) treatment, indicating that Dll4 induction on DCs is dependent on MyD88 signaling pathway as previously described [17]. When we examined BMDMs (CD11b^+^F4/80^+^), we found that Dll1 expression was induced during H1N1 stimulation as well as by PolyI:C (TLR3 ligand) and LPS stimulation, while no Dll4 expression was induced following any of these treatments (**Fig. 1D**). In addition, ELISA analysis showed that H1N1 stimulation as well as PolyI:C and LPS stimulation, but not CpG stimulation, induced production of type-I IFNs by BMDMs (**Fig. S2**). The increased gene expression of both Dll1 and IFN-β were also associated with an increase of the viral load of H1N1 (**Fig. S3**).

### Dll1 expression on BMDMs is dependent upon type-I IFN

**Figure 2:**
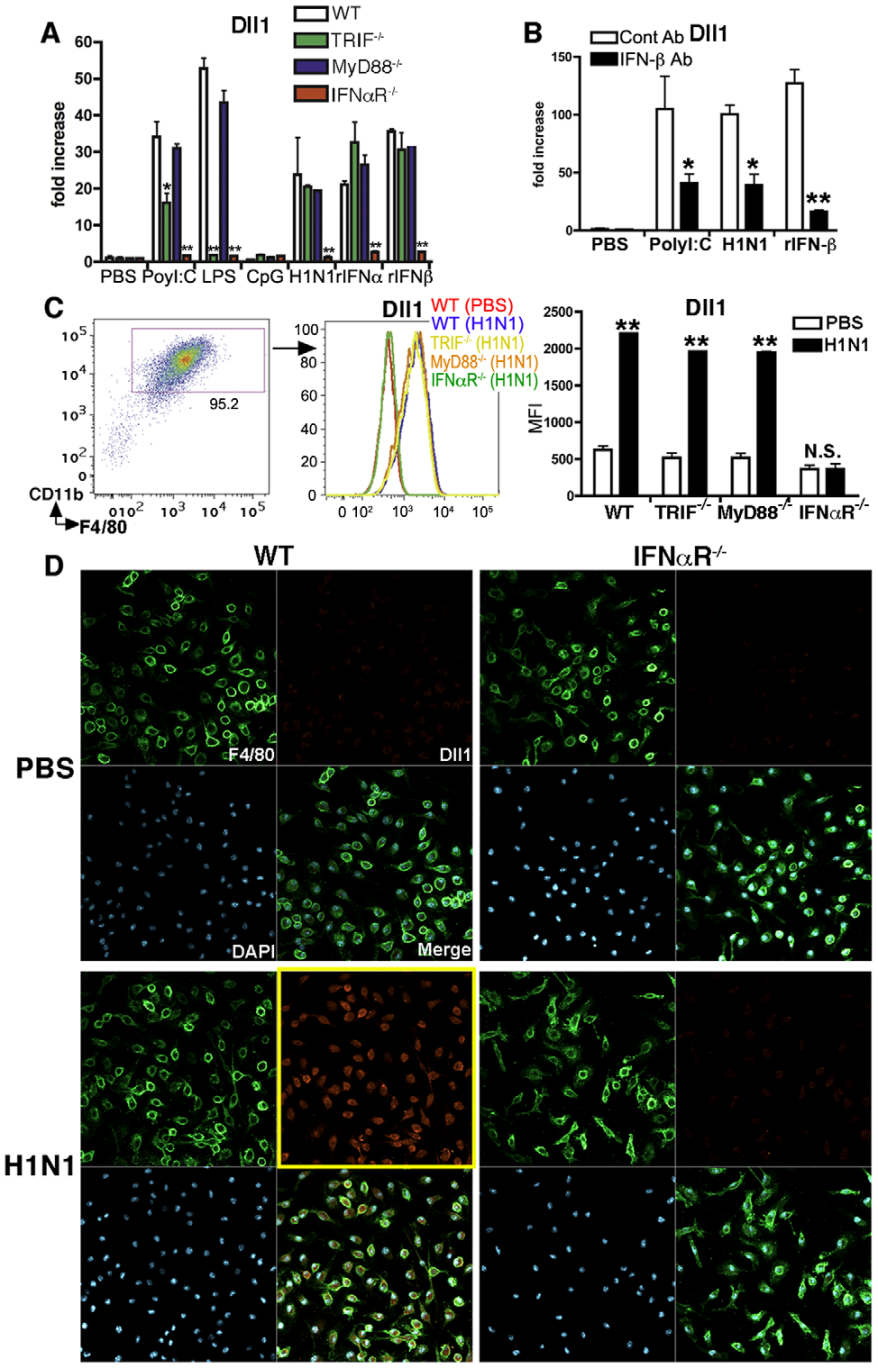
Dll1 expression expression is dependent upon type-I IFN. (**A**) Gene expression of Dll1 on BMDMs from WT, TRIF^−/−^, MyD88^−/−^, or IFNαR^−/−^ mice following stimulation with PolyI:C (10 µg/ml), LPS (1 μg/ml), CpG (1μM), H1N1 (MOI = 10), recombinant (r) IFN-α (20 Units), or rIFN-β (20 Units) for 6 hours. *P<0.05, **P<0.01 compared with WT mice. (**B**) The gene expression level of Dll1 on BMDMs pretreated with anti-mouse IFN-β Ab (2 µg/ml). *P<0.05, **P<0.01 compared with control (cont) Ab. (**C**) The level of Dll1 on BMDMs (CD11b^+^F4/80^+^) following stimulation with H1N1 (MOI = 10) for 24 hours determined by flow cytometry. **P<0.01 compared with PBS-treated BMDMs. (**D**) Confocal immunofluorescent examination of BMDMs stimulated with H1N1 (MOI = 10) between WT and IFNαR^−/−^ mice. F4/80: green, Dll1: red, DAPI: blue. Original magnification, ×400. Data shown are mean ± SEM and are a representative experiment of 3 independent experiments. Each point represents at least 3 mice per group.

To further investigate the induction mechanism for Dll1, we examined Dll1 expression using WT, TRIF^−/−^, MyD88^−/−^, and IFNαR^−/−^ mice. As shown in **Fig. 2A**, the mRNA expression levels of Dll1 following H1N1 stimulation in BMDMs from IFNαR^−/−^ mice was completely abrogated, while Dll1 expression in TRIF^−/−^ and MyD88^−/−^ mice was comparable to its expression in WT mice. Further, LPS stimulation of BMDMs from TRIF^−/−^ mice did not increase expression of Dll1 when compared to WT mice. Moreover, BMDMs from IFNαR^−/−^ mice had impaired induction of Dll1 mRNA following each stimulation condition we examined (**Fig. 2A**). Additionally, when BMDMs were pretreated with anti-IFN-β Ab before treatment with H1N1 and PolyI:C, the expression of Dll1 was significantly decreased (**Fig. 2B**). Flow cytometry data confirmed that Dll1 protein was not induced in BMDMs from IFNαR^−/−^ mice following H1N1 stimulation (**Fig. 2C**). These results were also supported by confocal immunofluorescent analysis, which indicated Dll1-positive expression (red) on F4/80-positive macrophages (green) following influenza virus treatment in WT mice; however, in IFNαR^−/−^ mice, F4/80-positive macrophages (green) were Dll1-negative (red) following H1N1 stimulation (**Fig. 2D**).

### RIG-I like pathway and IFNαR-JAK/STAT pathway are involved in Dll1 induction

**Figure 3:**
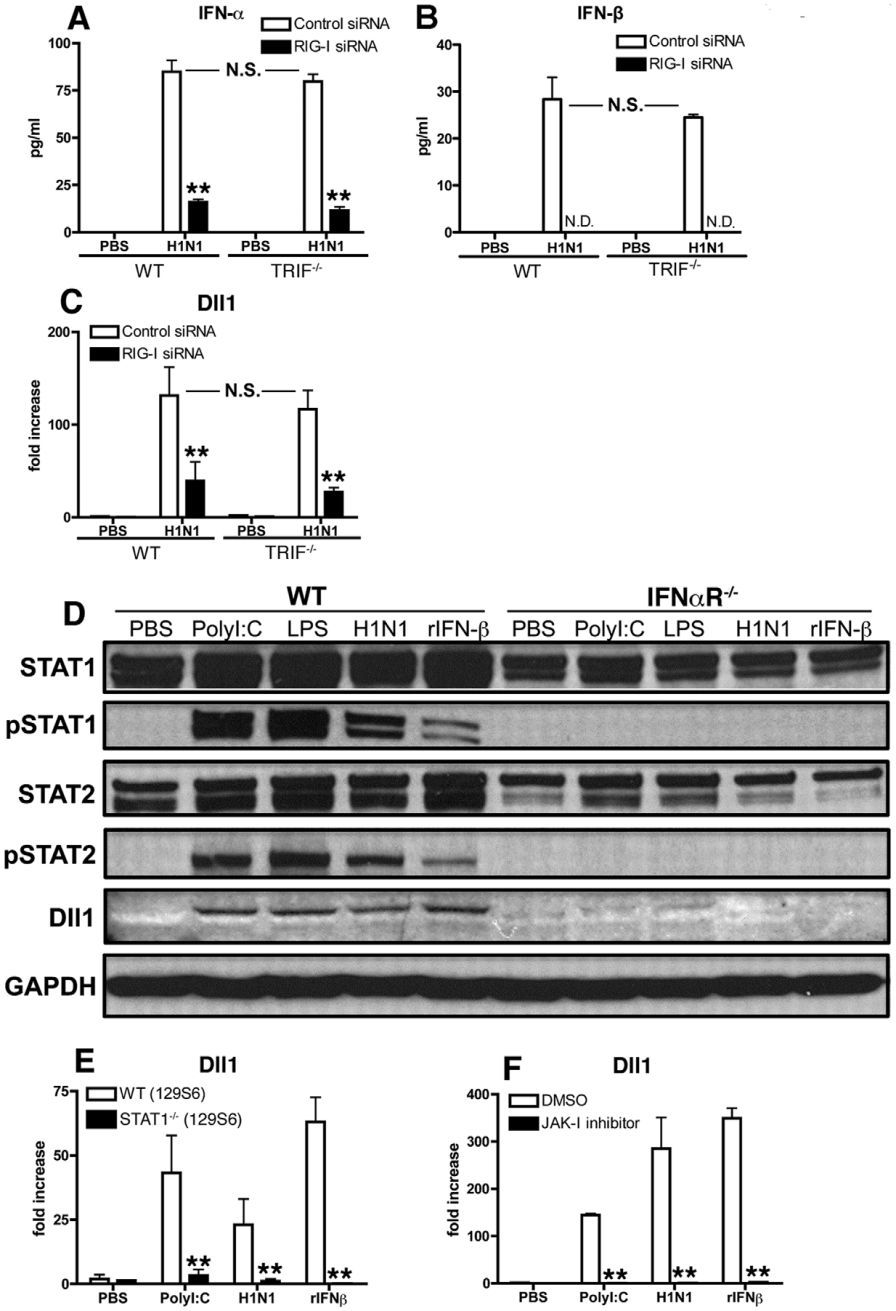
RIG-I- and JAK/STAT-dependent Dll1 induction in BMDMs. (**A**–**C**) BMDMs were transfected with RIG-I siRNA or control siRNA. The cells were then incubated with H1N1 for 24 hours (**A**, **B**) or for 6 hours (**C**), and the production of IFN-α (**A**) and IFN-β (**B**) was determined by ELISA, and the expression of Dll1 gene was measured by quantitative real-time PCR (**C**). **P<0.01 compared with control siRNA treated BMDMs. N.D.  =  not detectable (**D**) BMDMs were stimulated with PolyI:C (10 µg/ml), LPS (1 µg/ml), H1N1 (MOI = 10), or rIFN-β (20 Units) for 6 hours, and then expression of STAT1, phosphorylated (p)STAT1, STAT2, pSTAT2, and Dll1 were measured by western blotting. GAPDH was used as a loading control. (**E**, **F**) BMDMs were stimulated with PolyI:C (10 µg/ml), H1N1 (MOI = 10), or rIFN-β (20 Units) for 6 hours, and then Dll1 gene expression was analyzed by quantitative real-time PCR. (**E**) Dll1 expression on BMDMs between WT and STAT1^−/−^ mice. **P<0.01 compared with WT (129S6) mice. (**F**) Dll1 expression on BMDMs between DMSO and JAK-I inhibitor treatment (10 µM). **P<0.01 compared with DMSO treatment. Data shown are mean ± SEM and are a representative experiment of 3 independent experiments. Each point represents at least 4 mice per group.

RNA virus can trigger the TLR3-TRIF signaling pathway and/or the RIG-I like pathway, each of which induces type-I IFN. To determine whether these pathways also regulate Dll1 Notch ligand expression, we next examined type-I IFN production and Dll1 gene expression levels during H1N1 stimulation in TRIF^−/−^ mice or by knocking down the RIG-I gene. IFN-α protein levels were significantly lower and IFN-β protein was not detectable in RIG-I siRNA-treated BMDMs compared with control siRNA-treated BMDMs (**Fig. 3** **A** and **B**). In contrast, levels of type 1 IFN expression were unchanged when BMDMs from TRIF^−/−^ mice were compared to BMDMs from control mice (**Fig. 3** **A** and **B**). Similarly, the gene expression level of Dll1 was significantly lower in RIG-I siRNA-treated macrophages when compared with control siRNA-treated macrophages, whereas there was no significant difference in Dll1 gene expression between BMDMs from WT and TRIF^−/−^ mice (**Fig. 3C**). The above studies (**Fig. 2**) suggested that signaling through IFNαR is critical for Dll1 induction. Thus, we next examined the contribution of the JAK/STAT pathway, which is downstream to IFNαR activation, on Dll1 expression. Following PolyI:C, LPS, H1N1 or rIFN-β stimulation, both STAT1 and STAT2 were phosphorylated and Dll1 was detected in BMDMs from WT mice (**Fig. 3D**). However, in BMDMs from IFNαR-deficient mice no STAT1/2 phosphorylation and no Notch ligand Dll1 expression were seen. We also demonstrated that BMDMs from STAT1^−/−^ mice and BMDMs from WT mice treated with JAK-I inhibitor failed to induce the expression of Dll1 following stimulation with PolyI:C, H1N1 or rIFN-β (**Fig. 3** **E** and **F**). In addition, knocking down the STAT2 gene led to significantly lower expression of Dll1 (data not shown). Together these results suggest that phosphorylation of STAT1 and STAT2 are critical for Dll1 expression as is type-I IFN signaling through IFNαR. Thus, H1N1 infection leads to the production of type-I IFN via the RIG-I pathway in BMDMs. Type-I IFNs in turn bind to IFNαR in an autocrine loop and activates the JAK/STAT pathway that results in the transcription of Dll1.

### IFNαR^−/−^ mice are susceptible to influenza virus infection with impaired induction of Dll1

**Figure 4:**
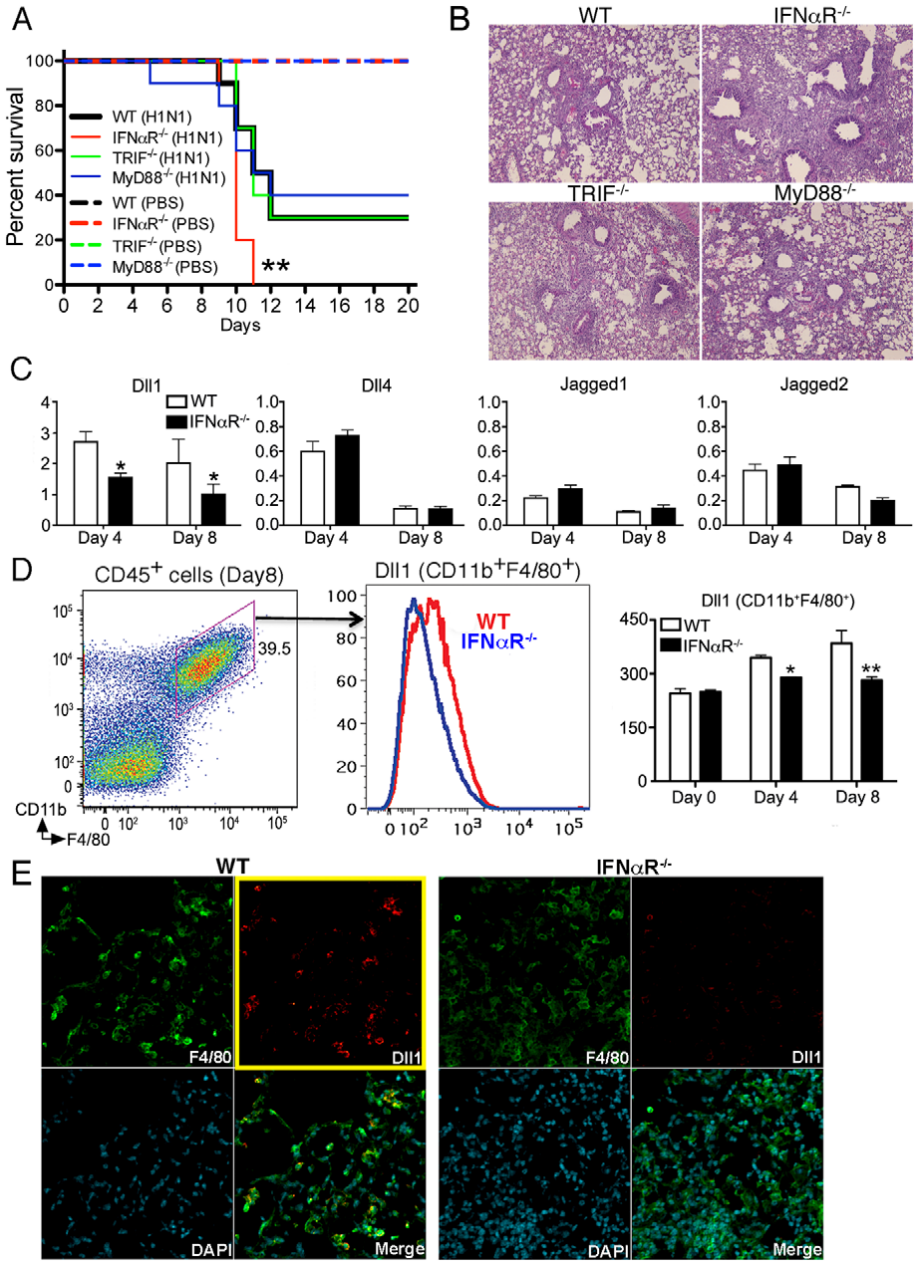
IFNαR ^−**/**−^ **mice showed higher mortality with impaired Dll1 expression during influenza virus infection.** (**A**) Survival rate in WT (black), IFNαR^−/−^ (red), TRIF^−/−^ (green), and MyD88^−/−^ (blue) mice. Mice were inoculated intranasally with PBS (dotted line) or H1N1 at 1×10^4^ PFU (solid line) per each group of mice. Results are expressed as the percentage of survival from 10 individual mice per group. **P<0.01 compared with WT mice (**B**) Histological appearance of lungs from WT, IFNαR^−/−^, TRIF^−/−^, MyD88^−/−^ mice at 8 days post-infection of influenza virus (**C**) Quantitative real-time PCR was performed to measure the transcript levels of Notch ligands at Day 4 and Day 8 after inoculation of influenza virus. *P<0.05 compared with WT mice (**D**) The level of Dll1 in lung macrophages (CD11b^+^F4/80^+^) from WT or IFNαR^−/−^ mice was determined by flow cytometry at Day 4 and Day 8 after inoculation of influenza virus. *P<0.05, **P<0.01 compared with WT mice. (**E**) Confocal immunofluorescent examination of influenza infected lungs at 8 days post-infection, Dll1^+^ cells (red) merged with F4/80^+^ cells (green) in WT and IFNαR^−/−^ mice. Blue staining indicates DAPI. Original magnification,×200. Shown are representative sections from 1 mouse of 4 per group. Data shown indicate mean ± SEM and are from a representative experiment of 3 independent experiments. Each time point represents at least 4 mice per group.

Because we showed that Notch ligand Dll1 was critically regulated by IFNαR in vitro, we next examined whether IFNαR^−/−^ mice infected with influenza virus failed to upregulate Dll1. First, we monitored the survival of WT, IFNαR^−/−^, TRIF^−/−^, and MyD88^−/−^ mice following H1N1 infection up to Day 20. We confirmed that the absence of IFNαR led to increased mortality after viral challenge when compared to WT mice (**Fig. 4A**). However, mice deficient for TRIF or MyD88 were not significantly different from WT mice regarding mortality (**Fig. 4A**). These findings were confirmed in lung histology studies 8 days post infection, that showed a significant increase in lung inflammation in IFNαR^−/−^ mice, as compared to the WT, TRIF^−/−^, and MyD88^−/−^ mice (Fig. 4B). In agreement with our in vitro BMDM data, we found that Dll1 mRNA levels were increased in whole lungs from WT mice over the study period, while the expression of Dll1 in whole lungs from IFNαR^−/−^ mice was significantly lower on both Day 4 and Day 8 after viral challenge (**Fig. 4C**). In contrast, no significant difference was observed in expression of Dll4, Jagged1, and Jagged2 in lungs from WT and IFNαR^−/−^ mice (**Fig. 4C**). Dll3 expression was below detection levels of our assay (data not shown). In addition, flow cytometry demonstrated that Dll1 expression on lung macrophages (CD11b^+^F4/80^+^) was significantly lower in IFNαR^−/−^ mice when compared with WT mice (**Fig. 4D**). These results were also confirmed by confocal microscopy, which showed impaired detection of Dll1 (red) on F4/80^+^ macrophages (green) in IFNαR^−/−^ mice during H1N1 infection (**Fig. 4E**).

### Macrophages play an essential role during influenza virus infection

**Figure 5:**
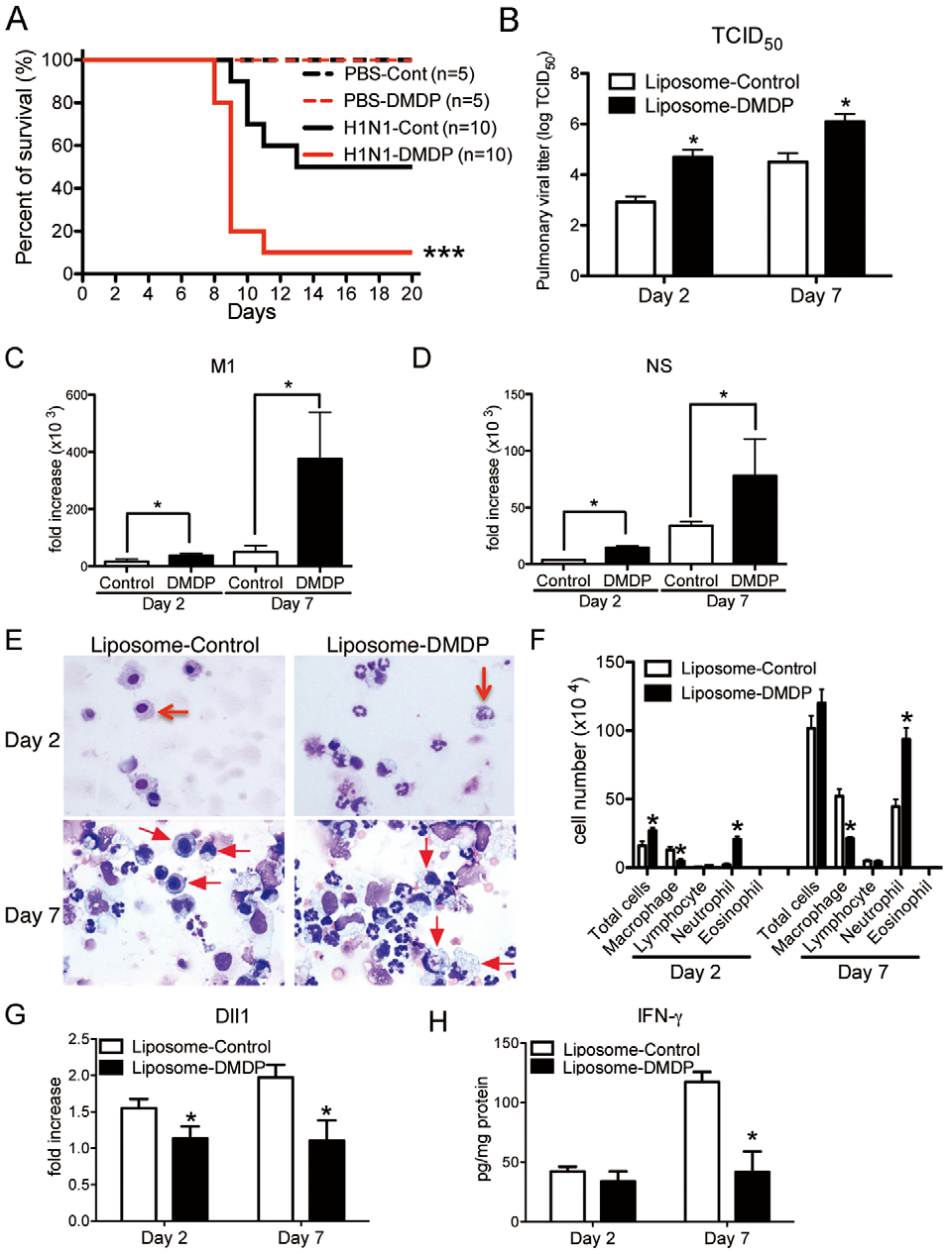
Macrophage is required for protection against influenza virus. Liposome-DMDP or liposome alone (20 µl/dose) was intranasally administrated to mice on day 1 and day 4 after influenza virus challenge. (**A**) Survival rate of WT mice treated with either control liposome (black) or liposome-DMDP (red) after intranasal injection with PBS only (dotted line) or H1N1 (solid line). ***P<0.001 compared with H1N1 group treated with control liposome. (**B**–**D**) Viral load in WT mice treated with either control liposome or liposome-DMDP at 2 and 7 days after infection of influenza virus (1×10^4^ PFU). (**B**) TCID_50_; Results are shown in log_10_ scale per lobe. M1 (**C**) and NS (**D**) H1N1 viral mRNA. Results are expressed as RNA copies normalized to GAPDH expression levels, as determined by real-time PCR. *P<0.05 compared with control liposome group. (**E**) Cellular cytospin appearance of bronchoalveolar lavage (BAL) harvested from mice treated with either control liposome or liposome-DMDP at day 2 and 7 after influenza virus infection stained with Giemsa stain. Red arrows show macrophages. Original magnification, ×1000. (**F**) The number of cells harvested from BAL. *P<0.05 compared with control liposome group. (**G**) Quantitative real-time PCR (Taqman) was performed to measure the transcript levels of Dll1 at day 2 and 7 after inoculation of influenza virus. *P<0.05 compared with control liposome group. (**H**) IFN-γ production from whole lungs of WT mice treated with either control liposome or liposome-DMDP at day 2 and 7 after influenza virus infection. Cytokine levels of IFN-γ were measured using a Luminex system. *P<0.05 compared with control liposome group. Data shown indicate mean ± SEM and are from a representative experiment of 2 independent experiments. Each time point represents 5 mice per group.

To directly examine the importance of macrophages, we used liposome- Dichloromethylenediphosphonic acid (DMDP) to deplete macrophages [18]. Intranasal administration of liposome-DMDP during influenza infection led to higher mortality (**Fig. 5A**) with greater virus load of 50% tissue culture infective dose (TCID_50_) at both day 2 and day 7 post-infection (**Fig. 5B**). The gene expressions of influenza H1N1 viral specific mRNA for matrix protein (M1) and nonstructural protein (NS) were also significantly higher in liposome-DMDP-treated mice (**Fig. 5** **C and D**). Cellular appearance of bronchoalveolar lavage (BAL) cells demonstrated decreased number of macrophages and increased number of neutrophils (**Fig. 5** **E and F**). In addition, most of the remaining macrophages in BAL cells from liposome-DMDP-treated mice that were counted in **Fig. 5F** had the appearance of dead cells. Moreover, the expression of Dll1 from whole lungs was significantly lower in liposome-DMDP-treated mice (**Fig. 5G**). We also demonstrated that protein levels of IFN-γ from lungs of macrophage depleted H1N1-infected mice were significantly impaired compared to control liposome-treated mice (**Fig. 5H**).

### Dll1 regulates immune response against influenza infection

**Figure 6:**
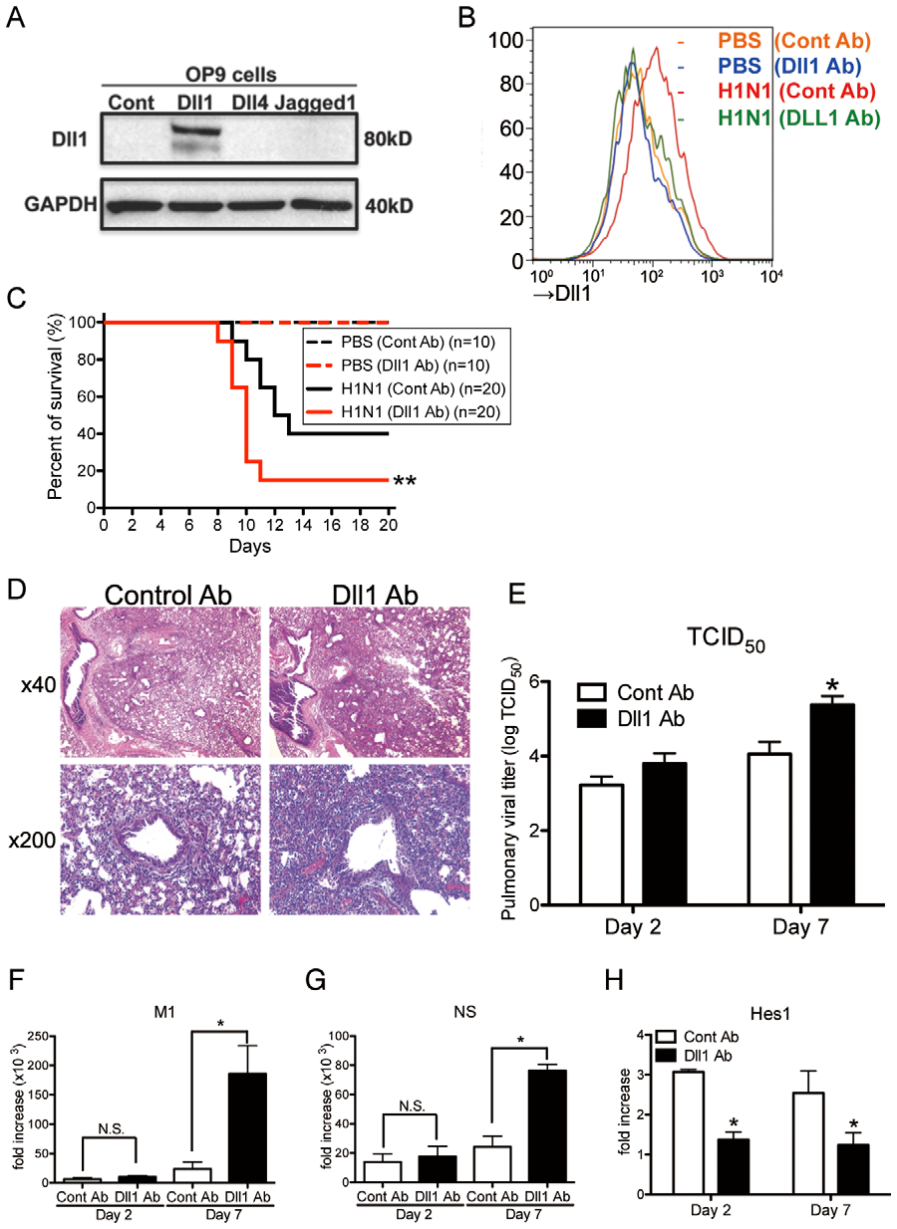
Passive immunization using Abs against Dll1 abrogates survival rate, lung pathology, and viral load. (**A**) Western blot analysis showed the specificity of polyclonal rabbit anti-Dll1 using OP9 cells transfected with various Notch ligands. (**B**) The level of Dll1 in lung macrophages (CD11b^+^F4/80^+^) was determined with flow cytometry using an Ab against Dll1 at day7 post infection; PBS treated mice with either control Abs (Orange) or Abs directed against Dll1 (Blue), H1N1-infected mice with either control Abs (Red) or Abs directed against Dll1 (Green). (**C**) Survival rate of WT mice treated with either control Abs (black) or Abs directed against Dll1 (red) after intranasal injection with PBS only (dotted line) or H1N1 (solid line). **P<0.01 compared with control Ab group. (**D**) Histological appearance of lungs isolated from WT mice treated with either control Abs or Dll1 Abs at day 2 and 7 after influenza virus infection. H&E staining. Original magnification, ×40; ×200. (**E–G**) Viral load in WT mice treated with control Abs or anti-Dll1 Abs at 2 and 7 days after infection of influenza virus (1×10^4^ PFU), measured by TCID_50_ (**E**) and, M1 (**F**) and NS (**G**) H1N1 viral mRNA. Viral mRNAs are expressed as RNA copies normalized to GAPDH expression levels, as determined by real-time PCR. (**H**) mRNA expression of Hes1 in mice treated with control Abs or anti-Dll1 Abs 2 and 7 days after infection of influenza virus. *P<0.05 compared with control Ab group. Data shown indicate mean ± SEM and are from a representative experiment of 3 independent experiments. Each time point represents 5 mice per group.

To directly test the effect of Dll1 against influenza infection, we blocked Dll1 functionality in WT mice by intraperitoneal passive immunization with anti-murine Dll1 Ab. We confirmed the specificity of this antibody with stably transfected OP-9 cell lines for Notch ligands Dll1, Dll4, or Jagged1. The purified antibody was found to react only with the cell line expressing Dll1 (**Fig. 6A**). Mice were treated intraperitoneally with anti-Dll1 or control IgG antibody (1 mg) on day 0, 2, and 4 of viral challenge. We also examined the expression of Dll1 from lung macrophages (CD11b^+^F4/80^+^) at Day 7 post-infection to demonstrate whether the Dll1 antibody has an inhibitory effect in vivo. Flow cytometry analysis showed that the protein level of Dll1 after treating H1N1 infected mice with anti-Dll1 antibody was similar to that seen in control PBS-treated mice (**Fig. 6B**). Treatment with this purified anti-Dll1 Ab led to significantly increased mortality compared to control IgG treated mice (**Fig. 6C**). Histological assessment showed more severe pneumonia in anti-Dll1-treated mice 7 days post influenza infection (**Fig. 6D**). Next, viral load was assessed by measuring both TCID_50_ (**Fig. 6E**) and influenza H1N1 viral specific mRNA for M1 and NS (**Fig. 6** **F and G**). The results showed significantly higher virus load in the lungs of mice that received anti-Dll1 Ab compared to controls at day 7 post-infection. We further demonstrated that the whole lung expression of Hes1, a downstream transcription factor which is a target of Notch pathways [17], was significantly lower in the lungs of H1N1 infected mice treated with anti-Dll1 Ab (**Fig. 6H**).

**Figure 7:**
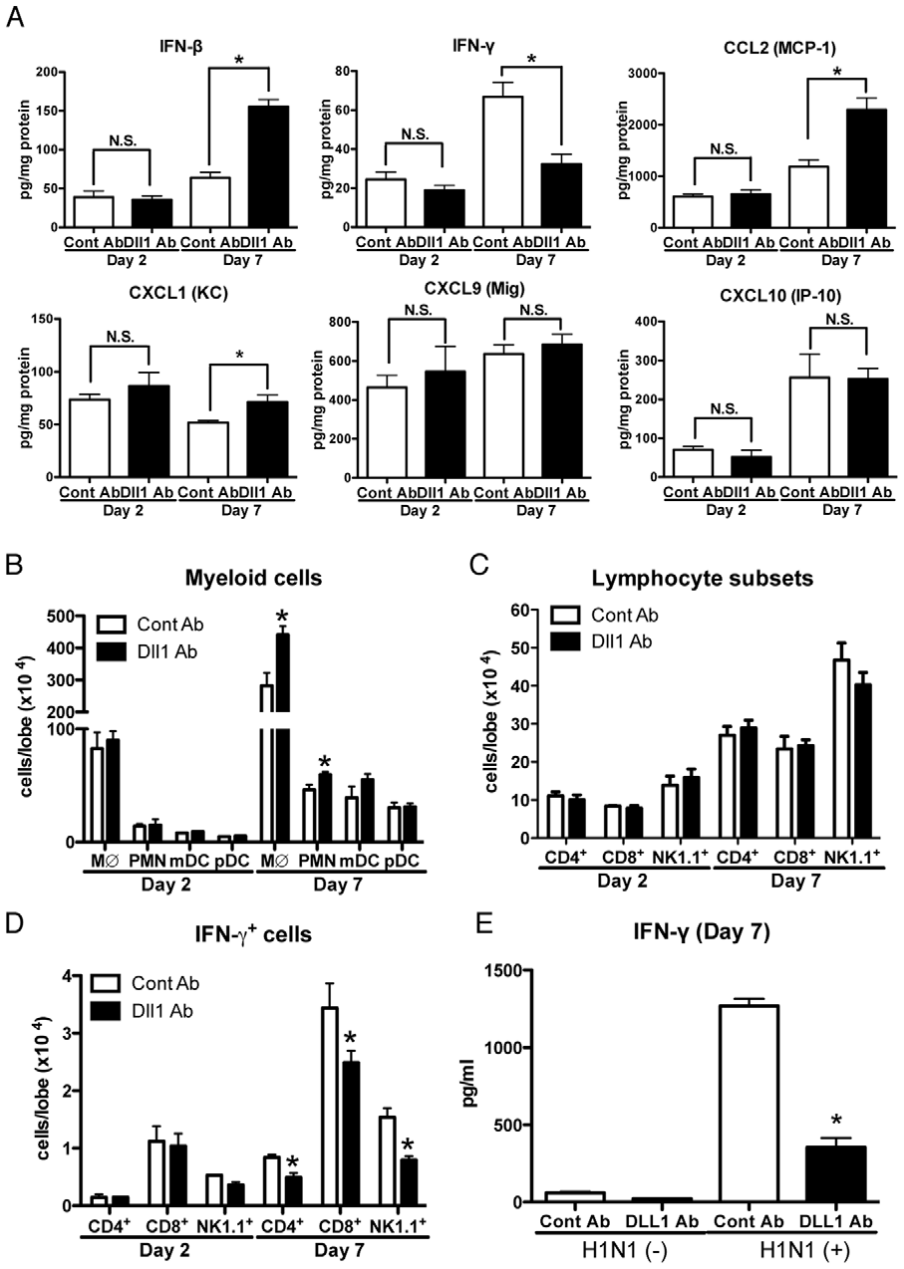
Blockade of Dll1 modulates immune response during influenza virus infection. (**A**) Protein levels of cytokines and chemokines in whole lungs isolated from WT mice treated with either control Abs or anti-Dll1 Abs at day 2 and 7 after influenza virus infection using a Luminex or ELISA system. *P<0.05 compared with control Ab group. (**B**) FACS analysis of lung macrophage (MØ; CD11b^+^F4/80^+^), neutrophil (PMN; Gr-1^high^), myeloid DC (mDC; CD11b^+^CD11c^+^), and plasmacytoid DC (pDC; B220^+^CD11c^+^) isolated from influenza challenged mice at day 2 and 7. *P<0.05 compared with control Ab group. (**C**) FACS analysis of lung T cell (CD4^+^, CD8^+^) and NK cell (NK1.1^+^) isolated from influenza challenged mice at day 2 and 7. (**D**) FACS analysis of intracellular staining of CD4^+^ cells, CD8^+^ cells, NK1.1^+^ cells for IFN-γ. *P<0.05 compared with control Ab group. (**E**) Whole cells from the draining lymph nodes of WT mice treated with either control Abs or anti-Dll1 Abs at day 7 after influenza virus infection were restimulated in vitro with H1N1 for 48 hours. Cytokine level of IFN-γ was measured using a Luminex system. *P<0.05 compared with control Ab group. Data shown indicate mean ± SEM and are from a representative experiment of 3 independent experiments. Each time point indicates at least 4–5 mice per group.

To help elucidate the mechanism underlying the increased mortality and severe inflammation seen in anti-Dll1 Ab treated mice, we examined the cytokine and chemokine profile in whole lungs during H1N1 challenge. Interestingly, the protein level of IFN-β was significantly higher while that of IFN-γ was significantly lower in H1N1-infected whole lungs from anti-Dll1-treated mice compared to lungs from control mice 7 days post-infection (**Fig. 7A**). Additionally, whole lungs from anti-Dll1 Ab treated mice at day 7 post-infection had significantly higher protein levels of CCL2 and CXCL1 (**Fig. 7A**), molecules that play a critical role in the recruitment of monocytes/macrophges and neutrophils into inflammatory lesions. The production of CXCL9 and CXCL10, which support the migration of Th1 cells, was similar in anti-Dll1-treated mice and control mice (**Fig. 7A**). In agreement with the chemokine and cytokine profile from whole lungs, flow cytometry demonstrated enhanced macrophage and neutrophil recruitment during H1N1 infection in anti-Dll1-treated mice at day 7 post-infection (**Fig. 7B**). There was no significant difference in the number of T cells (CD4^+^ and CD8^+^ cells), NK cells (NK1.1^+^), myeloid DCs (mDCs; CD11b^+^CD11c^+^), and plasmacytoid DCs (pDCs; B220^+^CD11c^+^) (**Fig. 7** **B** and **C**), whereas the number of IFN-γ^+^ cells from each subset (CD4^+^, CD8^+^, and NK1.1^+^) was significantly lower in anti-Dll1-treated mice (**Fig. 7D**). Cells recovered from draining lymph nodes of H1N1-infected mice after in vitro H1N1 rechallenge, also demonstrated significantly impaired production of IFN-γ compared to control treated mice (**Fig. 7E**).

### Blocking of Notch signaling abrogates pathogenesis of influenza virus infection

**Figure 8:**
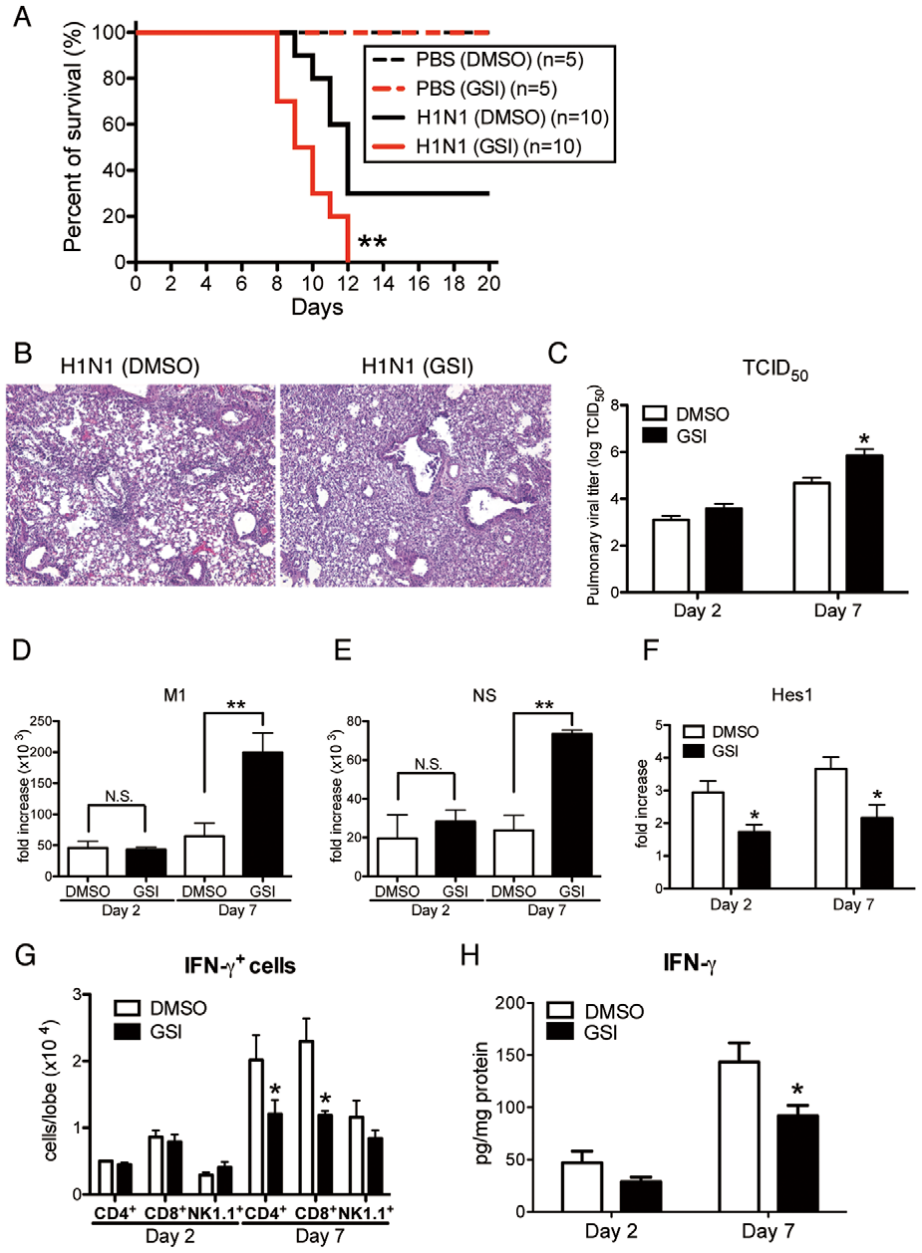
Blocking of Notch signaling abrogates survival rate, lung pathology, and viral load. GSI (10 nM; 50 µl volume) was intranasally administrated to mice on day 1 and day 4 after influenza virus challenge. 20% DMSO in 50 µl was used as a control for GSI. (**A**) Survival rate of WT mice treated with either control DMSO (black) or GSI (red) after intranasal injection with PBS only (dotted line) or H1N1 (solid line). **P<0.01 compared with H1N1 injection group treated with DMSO (**B**) Histological appearance of lungs isolated from WT mice treated with either control DMSO or GSI at day 7 after influenza virus infection stained with H&E. Original magnification, ×100. (**C–E**) Viral load in WT mice treated with DMSO or GSI at 2 and 7 days after infection of influenza virus (1×10^4^ PFU) measured by TCID_50_ (**C**); Results are shown in log_10_ scale per lobe. H1N1 viral mRNAs, M1 (**D**) and NS (**E**), were measured. Results are expressed as RNA copies normalized to GAPDH expression levels, as determined by real-time PCR. **P<0.01 compared with H1N1 injection group treated with DMSO. (**F**) mRNA exprerssion of Hes1 from whole lungs. (**G**) FACS analysis of intracellular staining of CD4^+^ cells, CD8^+^ cells, NK1.1^+^ cells for IFN-γ. (**H**) Cytokine level of IFN-γ from whole lungs was measured using a Luminex system. *P<0.05 compared with H1N1 injection group treated with DMSO. Data shown indicate mean ± SEM and are from a representative experiment of 2 independent experiments. Each time point represents 4**–**5 mice per group.

To directly examine the contribution of Notch signaling during influenza virus infection, we blocked Notch signaling by using GSI, a Notch signaling inhibitor. Intranasal administration of GSI during influenza infection led to higher mortality with excessive inflammation in the lungs compared to the control DMSO-treated group (**Fig. 8** **A and B**). Furthermore, viral load assessed by measuring both TCID_50_ and influenza H1N1 viral specific mRNA for M1 and NS indicated significantly higher virus load in the lungs of mice that received GSI compared to DMSO controls at day 7 post-infection (**Fig. 8** **C–E**). In addition, the expression of Hes1 from whole lung was significantly lower in the lungs of H1N1 infected mice treated with GSI (**Fig. 8F**). We also demonstrated significantly impaired production of IFN-γ from lung CD4^+^ and CD8^+^ T cells compared to control treated mice (**Fig. 8G**). Moreover, the data illustrate that there were considerable decreases in IFN-γ production of lungs from GSI-treated mice compared with control DMSO-treated mice (**Fig. 8H**).

### IFN-γ production from both CD4^+^ and CD8^+^ T cells during the immune response to H1N1 is optimized by co-culture with lung-derived macrophages

**Figure 9:**
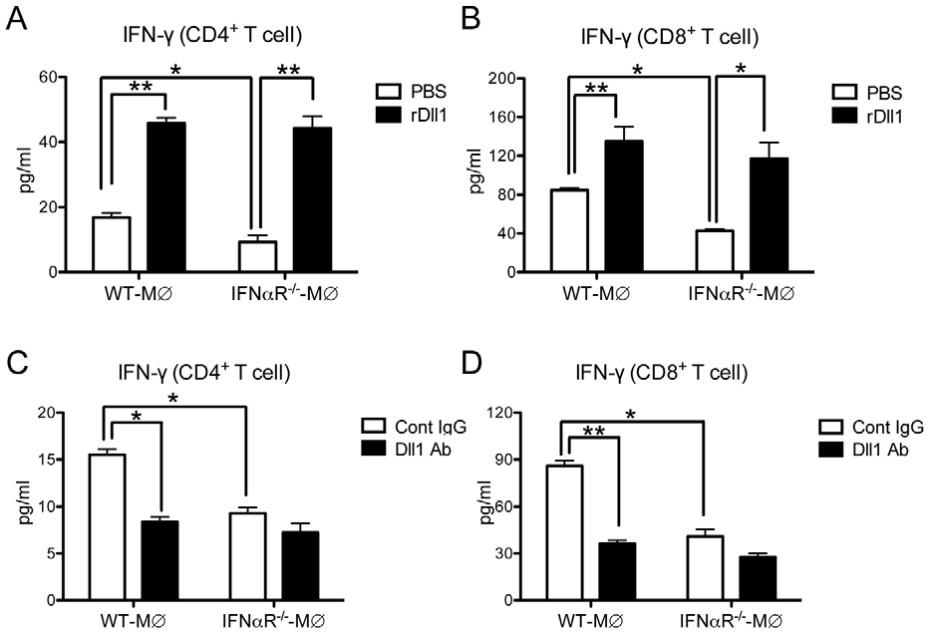
Activation of IFN-γ from lung T cells by lung macrophages during immune responses. (**A, B**) Lung CD4^+^ (**A**) or CD8^+^ (**B**) T cells were isolated from influenza virus challenged WT mice and stimulated with H1N1-pulsed lung-derived macrophages from either WT or IFNαR^−/−^ mice. Cells were co-cultured with plate-coated rDll1 (2.5 µg/ml) or PBS control. (**C, D**) Lung CD4^+^ (**C**) or CD8^+^ (**D**) T cells were isolated from influenza virus challenged WT mice and stimulated with H1N1-pulsed lung-derived macrophages from either WT or IFNαR^−/−^ mice. Cells were co-cultured with control IgG or anti-Dll1 Ab (20 µg/ml). Cytokine level of IFN-γ was measured using a Luminex system. Data shown are mean ± SEM and are from a representative experiment of 3 independent experiments. Each time point represents 4 mice per group. *P<0.05, ** P<0.01.

To directly test the effect of Dll1 on the T cells, we performed an in vitro lung CD4^+^ and CD8^+^ T cell cytokine expression assay with H1N1-stimulated lung-derived macrophages from either WT or IFNαR^−/−^ mice with either addition or deletion of Dll1. As shown in **Fig. 9** **A** and **B**, lung macrophages from the IFNαR^−/−^ mice caused a significant decrease in IFN-γ production by T cells when compared to co-cultures with WT macrophages (white bars). Moreover, addition of recombinant (r) Dll1 augmented IFN-γ production from T cells isolated from H1N1-challenged lungs and co-cultured with H1N1-treated lung-derived macrophages. The levels of IFN-γ using macrophages from IFNαR^−/−^ or WT mice were comparable in presence of rDll1. Additionally, anti-Dll1 Ab significantly decreased IFN-γ production from both CD4^+^ and CD8^+^ T cells (**Fig. 9** **C** and **D**). These responses were also seen when using both CD4^+^ and CD8^+^ T cells from draining lymph nodes (**Fig. S4**).

To verify that the addition of Dll1 to co-cultures of macrophages and T cells was activating Notch pathways, we used quantitative real-time PCR to examine Hes1 expression. Cultures receiving Dll1 showed a 3.60±0.45-fold increase in Hes1 expression over cultures with macrophages and T cells alone. Taken together, our findings suggest that Dll1 is able to skew T cell maturation via Notch signaling pathways.

## Discussion

Our results demonstrate that the Notch signaling pathway and, in particular, the Notch ligand Dll1 is essential in the regulation of influenza H1N1 virus infection. To our knowledge, this is the first report defining this relationship and delineating the underlying mechanisms. Of the five Notch ligands, Dll1 is the only Notch ligand specifically upregulated on macrophages following influenza stimulation, but it is not expressed on DCs. Also, the peak expression of Dll1 on lung macrophages in mice coincides with the period of peak inflammation after H1N1 infection. Our studies confirmed that lung macrophages from in vivo H1N1 infected mice expressed Dll1. Blocking Dll1 during viral infection led to significantly higher mortality and greater accumulation of inflammatory cells in the respiratory tract. In addition, neutralization of Dll1 during H1N1 infection altered CD4^+^ and CD8^+^ T cell activation responses as measured by IFN-γ^+^ producing cells within the lung. Together, these results have detailed the mechanisms by which the elements of the immune system cooperate and coordinate their efforts to eliminate viral infection. Our understanding of these mechanisms may possibly lead to clinical approaches to fight influenza pandemics.

The innate immune response is the first defense of the host to invading pathogens. Once initiated, proinflammatory cytokines and chemokines are released which cause macrophages and neutrophils to migrate to the source of infection [19]. Among the cytokines induced during the innate immune response, activation of type-I IFNs is the most powerful defense mechanism against influenza viral replication and spread [19]. We first demonstrated that macrophages, but not DCs, showed enhanced Notch ligand Dll1 expression in response to influenza virus and to type-I IFN cytokines, which suggested that Dll1 induction is dependent on type-I IFNs. We confirmed this by showing that IFNαR^−/−^-derived BMDMs completely failed to induce Dll1. Influenza virus amplifys the type-I IFN response via a positive-feedback loop that activates JAK-1 and Tyk-2 kinases, which leads to the phosphorylation and dimerization of STAT1 and STAT2 proteins [6], [20]. Our studies also showed impaired Dll1 induction on BMDMs from STAT1^−/−^ mice and BMDMs treated with a JAK-1 inhibitor. PRRs that recognize influenza virus RNA, have been shown to be a key initiator of type-I IFN response in infected cells [6]. These PRRs rely on the RIG-I-like signaling pathway, composed of RIG-I and MDA5, and also the TLR3-TRIF pathway [5]. Kato *et al.* demonstrated that mouse fibroblasts lacking RIG-I, but not MDA5, are defective in the production of type-I IFN in response to influenza virus [21]. Our study also showed that RIG-I-knocked down BMDMs expressed decreased Notch ligand Dll1 with significantly decreased type-I IFN cytokine production following influenza virus stimulation. We also observed that MDA5*-*knocked down BMDMs expressed levels of Dll1 similar to BMDMs treated with control siRNA (data not shown). In addition, we showed that Dll1 and type-I IFN production in BMDMs was TRIF independent. Thus, our results show that influenza virus-induced type-I IFNs are exclusively RIG-I dependent and that their production is essential for the induction of Dll1 through the IFNαR and the JAK-1/STAT1/2 signaling pathway.

Using IFNαR^−/−^ mice, our studies confirmed how critical type-I IFNs are for protection against influenza H1N1 virus in agreement with a recent report using influenza H5N1 virus [22]. Using a liposome-DMDP system [18], we also demonstrated that macrophages are indispensable for combating influenza virus infection. Though the depletion of macrophage seems incomplete from the number of macrophages remaining, most of the remaining macrophages in BAL cells from liposome-DMDP-treated mice that were counted were likely under going apoptosis. Interestingly, the production of type-I IFNs from whole lungs during influenza virus infection was higher in anti-Dll1-treated mice compared with control Ab-treated mice, suggesting that enhanced type-I IFNs production from anti-Dll1-treated mice might be due to impaired viral clearance. These findings indicate that Dll1 expression on macrophages is crucial for protection against influenza virus.

The initial interaction between invading microorganisms and the innate immune system critically influences the development of adaptive antiviral immunity [13]. Although both types of IFNs (type-I and type-II) play crucial roles in the immediate innate cellular response to viral infection, the immunomodulatory activities of IFN-γ have a large role in coordinating the adaptive immune response and in maintaining an antiviral state for longer times [12]. In addition, there is increasing evidence that the Notch system is an important bridge between APCs and T cell communication circuits [14], [15]. Other studies have demonstrated that APCs encountering pathogens that skew the immune response to a CD4^+^ Th1 cell response, showed an upregulation of Dll1 [14], [23]. Notch signaling is also associated with the differentiation of naive CD8^+^ T to cytotoxic T lymphocytes (CTLs) [24]. We first demonstrated that depletion of macrophages, a key player in Dll1 induction, induced decreased production of IFN-γ from lung CD4^+^ and CD8^+^ T cells with dampening of Dll1 levels during influenza virus infection. The change in Dll1 expression in this model was minor and suggested that we examined Dll1 expression in whole lungs. However, the upregulation of Dll1 returns to naïve mice levels in the absence of macrophages. Our results further showed that specifically blocking Dll1 during influenza infection impaired the survival and inflammatory status in our model with a decreased number of IFN-γ^+^CD4^+^ and IFN-γ^+^CD8^+^ T cells. Moreover, blocking of Notch signaling by GSI, which has been used in clinical trials as a cancer therapy approach, abrogated the survival and pathogenesis of lung inflammation with a decreased number of IFN-γ^+^CD4^+^ and IFN-γ^+^CD8^+^ T cells, suggesting the pivotal role of Dll1 through Notch signaling in driving IFN-γ mediated immune response to influenza virus. The expression of Hes1 in lungs was upregulated following influenza virus infection, and the treatment with anti-Dll1 antibody or GSI led to a decreased expression of Hes1. However, the reduction of IFN-γ from the lungs of influenza-infected mice with these treatments was approximately 30%. This incomplete reduction might be attributed to the immunity of NK cells, one of major producer of IFN-γ during influenza virus infection, to these treatments.

APCs, in particular, DCs and macrophages, have a key role in regulating and modulating the immune response [25]. Our findings indicated that induction of Dll1 on macrophages in response to influenza virus specifically regulated IFN-γ production from CD4^+^ and CD8^+^ T cells both in vivo and in vitro. Our studies demonstrate that anti-Dll1-treated mice exhibited significantly impaired survival accompanied by an impaired IFN-γ level. Our studies also showed that Dll1 is required for optimal IFN-γ production in response to Ag. Moreover, we demonstrated that GSI-mediated inhibition of Notch signaling attenuated overall IFN-γ production and resulted in fewer numbers of IFN-γ^+^CD4^+^ and IFN-γ^+^CD8^+^ T cells in our influenza model. Although IL-12 is known to be a strong inducer of CD4^+^ Th1 cell development, it has been reported that the Th1 response induced by Dll-mediated Notch signaling is IL-12 independent [26]. In our studies, blocking of IL-12 did not alter IFN-γ production from CD4^+^ and CD8^+^ T cells in co-culture system of APCs and T cells, and we could not detect IL-12 production in either BMDM or influenza virus-infected lungs (data not shown). Thus, our results show that Notch ligand Dll1 is required to promote IFN-γ production from CD4^+^ and CD8^+^ T cells in IL-12 independent manner, a scenario which might be important in the protective immune response against influenza virus.

Several studies support our results showing that IFN-γ plays an important role in recovery from influenza viral infection by helping to clear the virus [27], [28], [29]. In contrast, using IFN-γ-deficient mice, Graham *et al.* showed that IFN-γ is not necessary for recovery from influenza virus infection [30]. Possibly, the protective or non-protective role of IFN-γ is dependent on the model system. There may be a balance that is perturbed in some models that inhibits the protective effects of IFN-γ during viral infection. Certainly, given the pleotrophic effects of IFN-γ in the immune response, it is easy to envision that IFN-γ KO mice would experience many different signaling pathway perturbances, masking the protective effects of IFN-γ in a “normal” immune response to virus infection. Thus, in different models an imbalance between inhibitory and activating signals could determine the role of IFN-γ after influenza virus infection, with full activation and signaling through Dll1 overcoming influenza viral-induced-inhibition of IFN-γ. This is also in agreement with the known protective role for protease-activated receptor-2 against influenza virus via IFN-γ dependent pathway [29]. We have not evaluated these ideas in our model and further investigations are needed. Also, lung epithelial cells and fibroblasts play critical roles in influenza infectious models. However, Dll1 expression was not upregulated following H1N1 influenza stimulation in lung epithelial and fibroblast cell lines (data not shown). It is not known what role the Notch system plays in these cells during influenza infection; determining this also may reveal a potential clinical target for fighting influenza virus-induced pneumonia.

Neutrophils and macrophages are the dominant leukocytes recruited to the lung during an influenza infection [31], [32], and this process is markedly augmented in both IFNαR^−/−^ mice (data not shown) and WT mice treated with anti-Dll1 Ab. The recruitment of more inflammatory cells into lungs enhances damage to lung cells and structures, including the respiratory epithelium, which might be related to higher mortality. Importantly, we found significantly higher levels of chemokines CXCL1 and CCL2 in infected IFNαR^−/−^ mice (data not shown) and WT mice treated with anti-Dll1 Ab. CXCL1 plays a role in the recruitment of neutrophils, and CCL2 plays a role in macrophage recruitment [33]. It has been previously reported that blocking expression of CXCR2, the receptor for CXCL1, resulted in a reduction of neutrophil influx with prolonged host survival during influenza infection [34]. In addition, Dawson *et al.* showed that CCR2 deficiency, a major receptor for CCL2, leads to a milder inflammatory response with reduced lung pathology and increased survival rates because of defective macrophage recruitment [35]. The above published reports agree with our findings, which show that higher CXCL1 and CCL2 levels in both lungs from IFNαR^−/−^ mice and lungs with anti-Dll1 Ab might be correlated with not only enhanced neutrophil and macrophage migration into lungs but also impaired survival rate.

In summary, we present a comprehensive analysis of Notch ligand Dll1 participation in an infectious model of influenza H1N1 virus. Blockage of Dll1 resulted in accelerated inflammatory responses and decreased IFN-γ levels from CD4^+^ and CD8^+^ T cells during influenza infection. Macrophages are indispensable for the protection against influenza virus by their enhancement of Dll1 expression levels during infection. Furthermore, Dll1 expression on macrophages was specifically regulated by type-I IFN. This study supports the concept that an understanding of Notch signaling, especially Dll1 regulation, in the immune response to influenza virus can provide mechanistic approaches that may have clinical applicability.

## Materials and Methods

### Ethics statement

This study was carried out in strict accordance with the recommendations in the Guide for the Care and Use of Laboratory Animals of the National Institutes of Health. The protocol was approved by the University Laboratory Animal Medicine (ULAM) Facility at the University of Michigan Medical School. All animal protocols were approved by ULAM and all efforts were made to minimise suffering.

### Mice

WT C57BL/6 mice, WT 129S6 mice, and STAT1^−/−^ mice (129S6 Background) were purchased from Taconic. C57BL/6 mice lacking the IFNαR gene (IFNαR^−/−^) were provided by M. Kaplan (University of Michigan Medical School). All mice, including female MyD88^−/−^ and TRIF^−/−^ C57 BL/6 mice, were housed in the University Laboratory Animal Medicine (ULAM) Facility at the University of Michigan Medical School as described before [36]. All mice were used for experiments at 8–12 week of age. Age- and sex-matched mice were used in these studies.

### Reagents

Rat mAbs specific for mouse CD3 (17A2), CD4 (L3T4), CD8 (53–6.7), CD11b (M1/70), CD11c (HL3), CD16/32 (2.4G2), CD45 (30-F11), CD45R/B220 (RA3-6B2), Gr-1 (RB6-8C5), NK1.1 (PK136), MHC Class II (M5/114.15.2), IL-12 (C17.8), and IFN-γ (XMG1.2) were purchased from BD PharMingen. Rat Anti-F4/80 (CI: A3-1) mAb was purchased from Serotec. Hamster anti-Dll1 and anti-Dll4 mAb for flow cytometry were purchased from BioLegend. Antibodies to STAT1 and STAT2 were purchased from Cell Signaling Technology, and Millipore, respectively. PolyI:C was from InvivoGen. LPS from *Escherichia coli* (O55:B5) was from Sigma-Aldrich. Mouse cytosine-phosphate-guanosine (CpG) DNA was from Cell Sciences. Recombinant mouse IFN-α and IFN-β were from PBL InterferonSource. Mouse IFN-β Ab for neutralization was from BioLegend. JAK-I inhibitor and γ-secretase inhibitor (GSI) X, a cell-permeable hydroxyethylene dipeptide isostere that acts as a highly specific and a potent inhibitor of γ-secretase were from Calbiochem. DMDP encapsulated liposomes and control plain liposomes were from Encapsula. Mouse cell lines, RAW264.7, M2-10B4, and LA4 were purchased from the American Type Culture Collection (ATCC).

### Generation of rabbit anti-mouse polyclonal Dll1-specific antibody

Rabbit anti–mouse Dll1 antibodies were prepared by multiple-site immunization of New Zealand white rabbits with recombinant mouse Dll1 (R&D Systems) in CFA and boosted with Dll1 in IFA, as in previously described procedures from our laboratory [17]. Polyclonal antibodies were titered by direct ELISA against Dll1 coated 96-well plates and titered at 10^7^.

### Virus infection and sampling

Mice were sensitized by intranasal injection of 1.0×10^4^ PFU of influenza A virus strain (strain A/PR8/34; H1N1 isotype: ATCC) in 30 µl of PBS. PBS was inoculated intranasally into mock-infected mice. In some experiments, mice were treated intraperitoneally with anti-Dll1 or control IgG antibody (1 mg) on day 0, 2, and 4 of viral challenge. Lungs and mediastinal lymph nodes (LNs) were harvested at the indicated time after influenza infection. Lung left lobe was used for histological assessment, and each right lobe was used for the analysis of mRNA, protein, flow cytometry, and virus infectious titer. Lung homogenates were serially diluted in Minimum Essential Medium Eagle medium (Sigma-Aldrich) and virus infectious titers were measured using the 50% tissue culture infectious doses (TCID50) assay based on cytopathic effect as previously described [31].

### Histological and Immunofluorescent examination

Individual excised lung lobes were inflated and fixed with 10% buffered formalin for morphometric analysis. For immunofluorescent analysis, lungs were embedded in Tissue-Tek OCT compound, and then frozen in liquid nitrogen. Seven-micron cryostat sections were then fixed in ice-cold acetone, incubated with primary antibodies, followed by the addition of appropriate Alexa-labeled secondary reagents (Invitrogen Corp.). Finally, the sections were analyzed by Zeiss LSM 510 confocal microscope system (Carl Zeiss Inc.).

### Reverse Transcription and Real-time Quantitative PCR Analysis

Total RNA was isolated from the cultured cells and whole lungs using RNeasy Mini kit (Qiagen) following the manufacturer's instructions and was reverse transcribed in a 25μl volume. Briefly, 1.0 µg RNA was reverse transcribed to yield cDNA in a 25-µL reaction mixture containing 1× first strand (Life Technologies), 250 ng oligo(dT) primer, 1.6 mmol/L dNTPs (Invitrogen), 5 U RNase inhibitor (Invitrogen), and 100 U Moloney murine leukemia virus reverse transcriptase (Invitrogen) at 38°C for 60 min; and the reaction was stopped by incubating the cDNA at 94°C for 10 min. The SYBR primer sets for Notch lignads were purchased from Sigma-Aldrich [16]. Real-time quantitative PCR analysis was performed using an ABI 7700 sequence detector system (PE Applied Biosystems). The thermal cycling conditions included 50°C for 2 min and 95°C for 10 min, followed by 40 cycles of amplification at 95°C for 15 s and 55°C for 1.5 min for denaturing and annealing, respectively. Quantification of the genes of interests were normalized to GAPDH and expressed as fold increases over the negative control for each treatment at each time point, as previously described [16]. For virus quantification, cDNA was synthesized by using MultiScribe reverse transcriptase and random hexamers (PE Applied Biosystems) as previously described [37]. For Real-time quantitative PCR, the following SYBR primers were used: for the M1; forward 5’-CATCCCGTCAGGCCCCCTCA-3’, reverse 5’-GGGCACGGTGAGCGTGAACA-3’, for the NS; forward 5’-GGGGCAGCACTCTTGGTCTGG-3’, reverse 5’-CGCGACGCAGGTACAGAGGC-3’.

### Western blotting

BMDMs were lysed in lysis buffer (Cell Signaling), briefly sonicated, kept on ice for 30 minutes, and centrifuged at 15,000 g for 15 minutes. The supernatant was collected and stored at −80°C until use. Equal amounts (15–30 µg) of cell lysates were fractionated by sodium dodecyl sulfate–polyacrylamide gel electrophoresis (Invitrogen). Then the proteins were transferred onto a nitrocellulose membrane. After the overnight incubation with appropriate primary antibody, the membrane was counterstained with horseradish peroxidase-conjugated rabbit or mouse IgG antibody and visualized with enhanced chemiluminescence detection reagents (GE Healthcare).

### Short-interfering (si) RNA assay

A total of 1.5×10^6^ BMDMs were transfected with 2 µg of a mixture of RIG-I (Ddx58)-specific, MDA5 (Ifih1)-specific, STAT2-specific, or nontargeting control siRNAs (Dharmacon), using mouse macrophage nucleofector kit (Lonza) according to the manufacturer's instructions and plated in a 12-well plate. After 24 hours, cells were used for experiments.

### Protein analysis of cytokine

Murine cytokine and chemokine levels were measured in 50 µl samples using a Bio-plex bead-based cytokine assay purchased from Bio-Rad Laboratories. IFN-α and IFN-β levels were measured by ELISA according to manufacturer's instructions (PBL InterferonSource). The cytokine levels in lung homogenates were normalized to the protein present in cell-free preparation of each sample measured by the Bradford assay, as described previously [16].

### Flow cytometry

Flow cytometric analyses of lung cells were performed as previously described [16]. In brief, whole lungs were dispersed in 0.2% collagenase (Sigma-Aldrich) in RPMI 1640 (MediaTek) and 5% FBS (Atlas Biologicals) at 37°C for 45 minutes to obtain a single-cell suspension. The cells were stained with indicated Abs after 10 minutes of pre-incubation with CD16/CD32 Abs (Fc block) and fixed overnight with 4% formalin. For intracellular staining of cytokines, lung cells (1.0×10^6^ cells/well) were cultured in 48-well plates containing plate-bound anti-CD3 (5 µg/ml) and soluble anti-CD28 (2.5 µg/ml). After overnight incubation and in the presence of GolgiPlug (BD Biosciences — Pharmingen) for the last 2 hours at 37°C and 5% CO2, the cells were stained for surface markers with FITC-conjugated anti-CD4, anti-CD8, or anti-NK1.1 Abs, resuspended in fixation/permeabilization solution (BD Cytofix/Cytoperm Kit; BD Biosciences Pharmingen), and stained with PE-conjugated anti–IFN-γ Abs respectively. Cells were analyzed using a Cytomics FC 500 (Beckman Coulter), and data were analyzed by FlowJo software (Tree Star Inc.).

### Generation of BMDCs and BMDMs

BM was harvested from uninfected, normal mice, filtered through nylon mesh. For generation of BMDMs, BM cells were cultured in L929 cell-conditioned medium as described previously [16]. Six days after initial bone marrow culture, BMDM were transferred to well plates overnight. For generation of BMDCs, BM cells were seeded in T-150 tissue culture flasks at 10^6^ cells/ml in RPMI 1640-based complete media with GM-CSF 20 ng/ml (R&D Systems) after depletion of erythrocytes with lysis buffer. 6 days later, loosely adherent cells were collected and incubated with anti-CD11c coupled to magnetic beads for isolation of conventional DCs from the GM-CSF cultures (Miltenyi Biotec). The purity of CD11c was more than 94% using flow cytometry. The cells were plated in well plates overnight. The next day, macrophages and DCs were infected with certain stimuli.

### In vitro T cell treatments

7 days after 1.0×10^4^ PFU of H1N1 intranasal injection, CD4^+^ or CD8^+^ T cells from lungs or mediastinal LN were isolated using a magnetic bead column (Miltenyi Biotec). More than 95% of cells were CD4 or CD8 positive, respectively. For harvest of naïve lung macrophages, the whole lung cells dispersed in 0.2% collagenase were washed and resuspended in 10-ml RPMI 1640, and then incubated in a 100-mm cell culture dish for 2 hour at 37°C and the non-adherent cells were removed. Adherent cells were collected as lung macrophages, and more than 95% were F4/80 positive. Naïve lung macrophages were pulsed with H1N1 (MOI = 10) for 2 hours, and then T cells (2×10^5^ cells/well) were exposed to H1N1-pulsed lung macrophages in 96-well plates at APC:T cell ratio of 1∶5, and supernatants were harvested 48 hours later for cytokine protein analysis. Plate-bound recombinant Dll1 (rDll1) (R&D Systems) was used at a final concentration of 2.5 µg/ml, and anti-Dll1 Ab and control IgG were used at a final concentration of 20 µg/ml.

### Statistical analysis

Two-tailed Student's t test was performed in Prism (Graphpad) in all cases. of p<0.05 were considered statistically significant. *P<0.05; ***P 0.01; ***P<0.001.

## Supporting Information

Figure S1 **Mouse macrophage cell line, RAW 264.7 cells exhibit increased expression of Dll1.** RAW 264.7 cells were stimulated with PolyI:C (10 µg/ml), LPS (1 µg/ml), CpG (1 µM), or H1N1 (MOI = 10) for 6 hours, then quantitative real-time PCR was performed and the expression levels of Notch ligands Dll1 (**A**), Dll4 (**B**), Jagged1 (**C**), and Jagged2 (**D**) were evaluated. Dll3 expression was below detection levels of our assay.
(TIF)

Figure S2 **BMDMs produce type-I IFN following H1N1 as well as PolyI:C and LPS stimulation.** BMDMs were stimulated with PolyI:C (10 µg/ml), LPS (1 µg/ml), CpG (1 µM), or H1N1 (MOI = 10) for 24 hours, then cytokine levels of IFN-α (**A**) and IFN-β (**B**) from supernatants were measured by ELISA system.
(TIF)

Figure S3 **Influenza virus (H1N1) induces increased gene expression of Dll1 and IFN-β in dose-dependent manner.** BMDMs were stimulated with H1N1 (MOI = 0.1, 1.0, or 10.0) for 6 hours, then quantitative real-time PCR was performed and the expression levels of Dll1 (**A**) and IFN-β (**B**) were evaluated.
(TIF)

Figure S4 **Activation of IFN-γ from lung T cells by lung macrophages during immune responses induced by influenza virus.** (**A, B**) LN CD4^+^(**A**) or CD8^+^(**B**) T cells were isolated from influenza virus challenged WT mice and stimulated with H1N1-pulsed lung-derived macrophages from either WT or IFNαR^−/−^ mice. Cells were co-cultured with recombinant (r) Dll1 (2.5 µg/ml) or PBS control. (**C, D**) LN CD4^+^(**C**) or CD8^+^(**D**) T cells were isolated from influenza virus challenged WT mice and stimulated with H1N1-pulsed lung-derived macrophages from either WT or IFNαR^−/−^ mice. Cells were co-cultured with control IgG or anti-Dll1 Ab (20 µg/ml). Data shown are mean±SEM and are from a representative experiment of 2 independent experiments. Each time point represents 4 mice per group. *P<0.05, ** P<0.01.
(TIF)
