# Supplementary figures and images for: The Critical Role of Notch Ligand Delta-like 1 in the Pathogenesis of Influenza A Virus (H1N1) Infection
Source: PLoS Pathog. 2011 Nov 3;7(11):e1002341. doi: 10.1371/journal.ppat.1002341 (PMC3207886; doi:10.1371/journal.ppat.1002341)

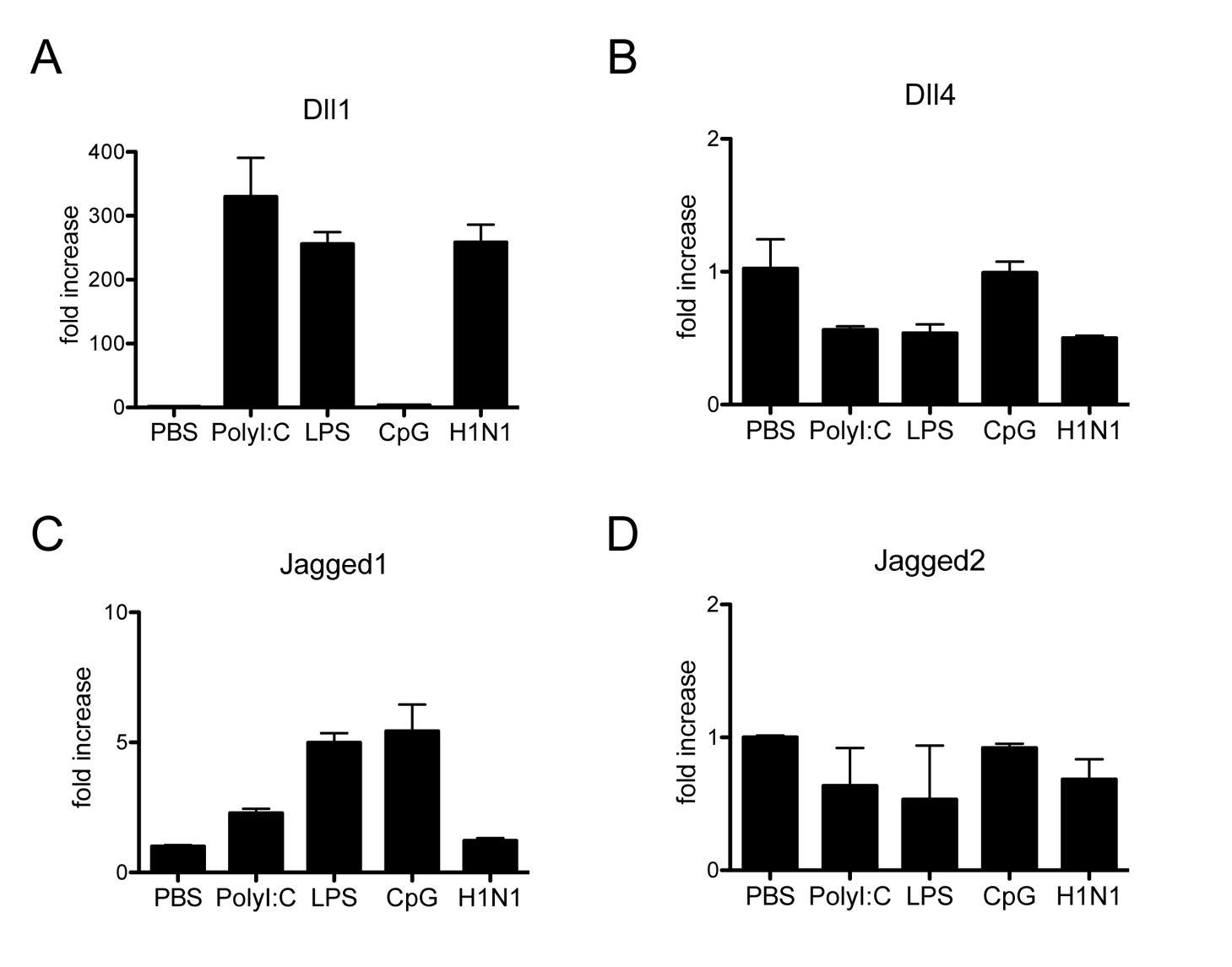

Supplement: Figure S1 — Mouse macrophage cell line, RAW 264.7 cells exhibit increased expression of Dll1. RAW 264.7 cells were stimulated with PolyI:C (10 µg/ml), LPS (1 µg/ml), CpG (1 µM), or H1N1 (MOI = 10) for 6 hours, then quantitative real-time PCR was performed and the expression levels of Notch ligands Dll1 (A), Dll4 (B), Jagged1 (C), and Jagged2 (D) were evaluated. Dll3 expression was below detection levels of our assay. (TIF) [file ppat.1002341.s001.tif]

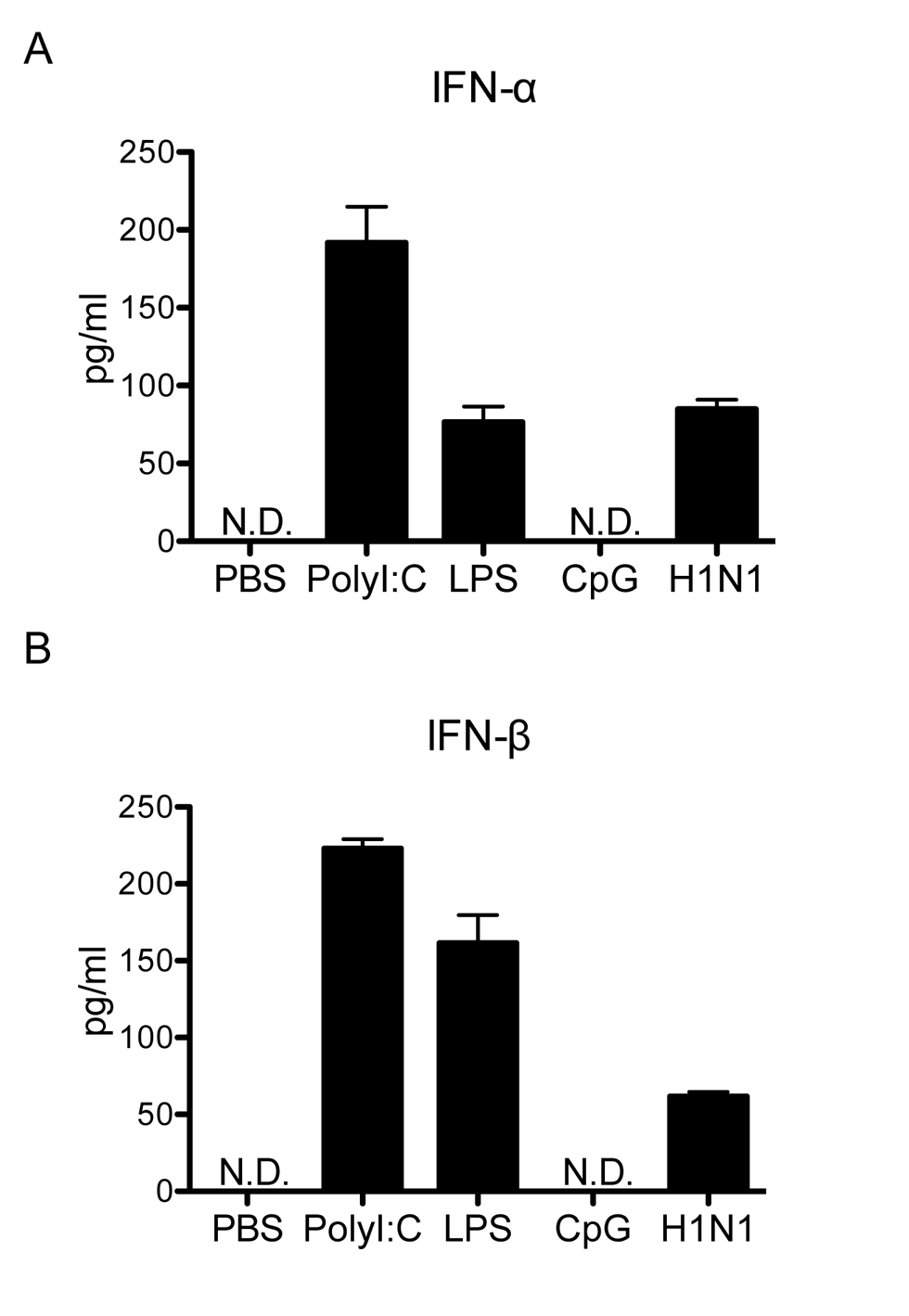

Supplement: Figure S2 — BMDMs produce type-I IFN following H1N1 as well as PolyI:C and LPS stimulation. BMDMs were stimulated with PolyI:C (10 µg/ml), LPS (1 µg/ml), CpG (1 µM), or H1N1 (MOI = 10) for 24 hours, then cytokine levels of IFN-α (A) and IFN-β (B) from supernatants were measured by ELISA system. (TIF) [file ppat.1002341.s002.tif]

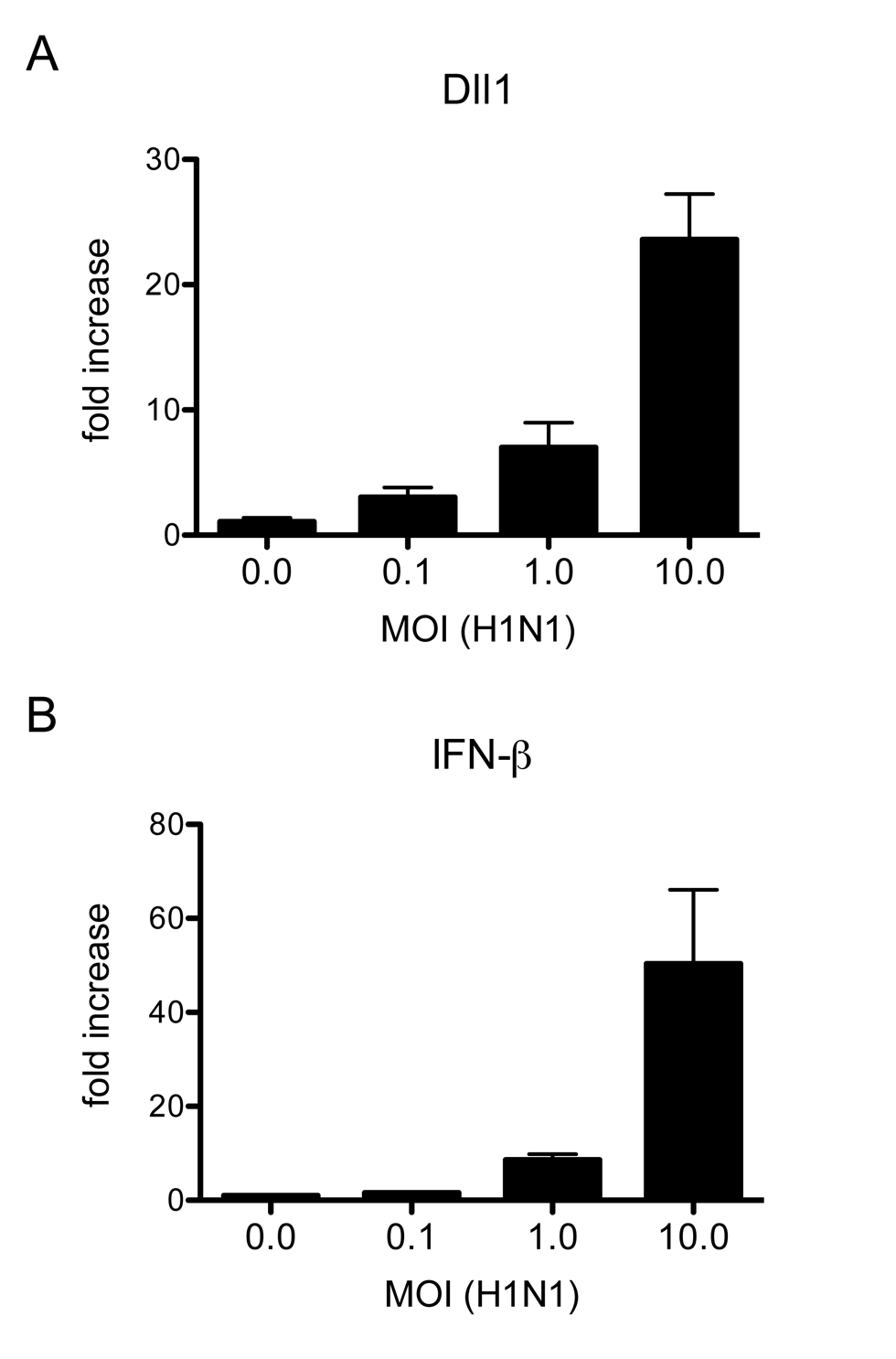

Supplement: Figure S3 — Influenza virus (H1N1) induces increased gene expression of Dll1 and IFN-β in dose-dependent manner. BMDMs were stimulated with H1N1 (MOI = 0.1, 1.0, or 10.0) for 6 hours, then quantitative real-time PCR was performed and the expression levels of Dll1 (A) and IFN-β (B) were evaluated. (TIF) [file ppat.1002341.s003.tif]

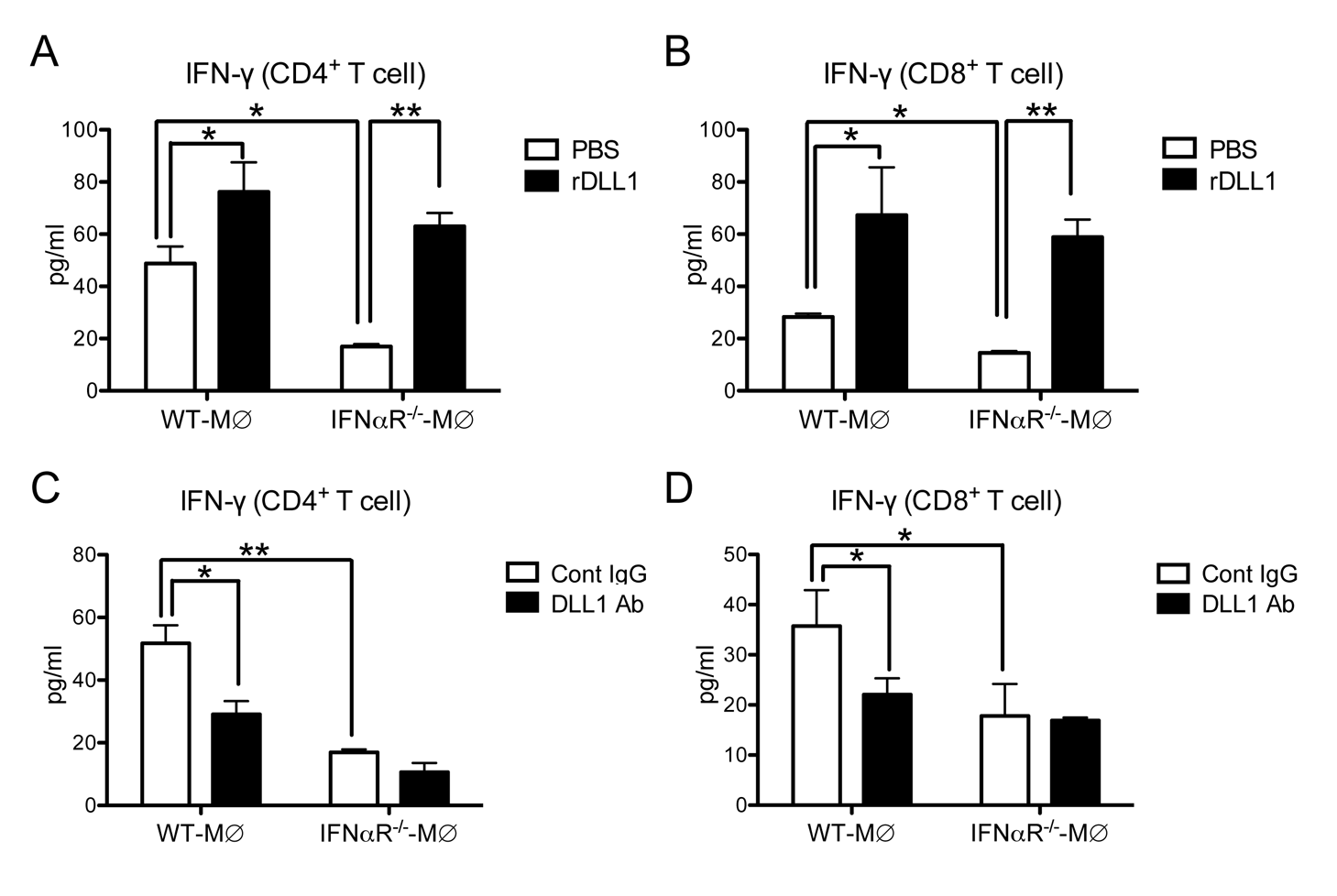

Supplement: Figure S4 — Activation of IFN-γ from lung T cells by lung macrophages during immune responses induced by influenza virus. (A, B) LN CD4+(A) or CD8+(B) T cells were isolated from influenza virus challenged WT mice and stimulated with H1N1-pulsed lung-derived macrophages from either WT or IFNαR−/− mice. Cells were co-cultured with recombinant (r) Dll1 (2.5 µg/ml) or PBS control. (C, D) LN CD4+(C) or CD8+(D) T cells were isolated from influenza virus challenged WT mice and stimulated with H1N1-pulsed lung-derived macrophages from either WT or IFNαR−/− mice. Cells were co-cultured with control IgG or anti-Dll1 Ab (20 µg/ml). Data shown are mean±SEM and are from a representative experiment of 2 independent experiments. Each time point represents 4 mice per group. *P<0.05, ** P<0.01. (TIF) [file ppat.1002341.s004.tif]
